# Integrative Multi-Omics Reveal Silibinin Alleviates Heat Stress-Driven Hepatic Lipid Disruption in Laying Hens

**DOI:** 10.3390/ijms27104267

**Published:** 2026-05-11

**Authors:** Jiang Gao, Hongrui Ren, Xuanfu Wu, Cunzhi Zou, Bin He, Wenqiang Ma

**Affiliations:** 1 Key Laboratory of Animal Physiology and Biochemistry, Ministry of Agriculture and Rural Affairs, College of Veterinary Medicine, Nanjing Agricultural University, Nanjing 210095, China; 2022007052@stu.njau.edu.cn (J.G.); heb@njau.edu.cn (B.H.); 2 Ministry of Education Joint International Research Laboratory of Animal Health & Food Safety, Nanjing Agricultural University, Nanjing 210095, China

**Keywords:** silibinin, heat stress, laying hen, antioxidant, liver lipid metabolism

## Abstract

Heat stress (HS) has emerged as a major environmental stressor, inducing oxidative stress and hepatic steatosis and impairing production performance and health in laying hens, with limited evidence-based nutritional interventions available. This study investigated the hepatoprotective effects of dietary silibinin (SIL) against chronic HS. In a 10-week trial, 252 43-week-old Hy-Line Brown hens were exposed to daily HS (32 ± 1 °C, temperature–humidity index [THI] > 73) and fed either a basal diet or one supplemented with 100 mg/kg SIL. SIL significantly increased laying rate (*p* < 0.05) and improved albumen height, Haugh units, and shell strength by week 8 (*p* < 0.05). Histological analysis showed a 48% reduction in non-alcoholic fatty liver disease (NAFLD) activity score, with significantly decreased hepatic triglyceride content (*p* < 0.05); Oil Red O staining confirmed reduced lipid droplet accumulation. SIL restored redox balance by increasing plasma, hepatic total superoxide dismutase (T-SOD), and glutathione peroxidase (GSH-Px) (*p* < 0.05), increasing hepatic catalase (CAT) and glutathione (GSH) levels while decreasing malondialdehyde (MDA) (*p* < 0.05). Untargeted plasma metabolomics identified 11 key metabolites related to 2-oxoglutarate and purine metabolism, while hepatic transcriptomics revealed 835 differentially expressed genes primarily in the PPAR signaling and fatty acid biosynthesis pathways. SIL suppressed de novo lipogenesis via downregulation of ACACA and FASN, and enhanced β-oxidation through upregulation of CPT1A and ACSL1 (*p* < 0.05). Molecular docking indicated favorable binding affinities between SIL and these targets, which was further supported by corresponding changes in protein expression via Western blotting. Correlation analysis revealed a consistent alignment between the upregulation of ACSL1/CPT1A and improvement in performance and antioxidant status, suggesting a coordinated metabolic shift. These findings emphasize the potential of SIL as a sustainable animal nutrition antioxidant additive, which can alleviate HS-induced lipid disorders in the liver of laying hens. Importantly, these hepatoprotective effects were demonstrated exclusively under chronic heat stress conditions; further studies incorporating a normothermic baseline are required to distinguish stress-specific mitigation from general metabolic stimulation.

## 1. Introduction

Climate change has intensified heat stress episodes, posing a major bottleneck for sustainable poultry production [1]. This results in economic losses exceeding billions of dollars annually from reduced productivity, morbidity, and mortality [2]. Among avian species, laying hens exhibit pronounced susceptibility owing to their elevated endogenous heat production, limited capacity for active evaporative thermoregulation, and the metabolic imperative of persistent oogenesis. Acute or chronic heat stress curtails feed intake by 15~30% and depresses productivity by 20~40%, while simultaneously derailing calcium and energy homeostasis and provoking oxidative damage, ultimately precipitating marked declines in laying performance and egg quality [3]. The hepatic organ serves as the primary locus mediating heat stress-induced metabolic dysregulation [4]. Hepatocytes integrate gluconeogenic, lipid metabolic, vitellogenic, and xenobiotic-detoxification pathways that are indispensable for ovarian follicular maturation and the biosynthesis of egg constituents [5]. Heat stress initiates hepatic steatosis, mitochondrial bioenergetic failure, and inflammatory infiltrates, which collectively compromise vitellogenin biosynthesis, yolk-precursor trafficking, and systemic metabolic efficacy [6]. Given the liver’s central role, interventions targeting metabolic stability are essential—yet conventional approaches fall short.

Conventional ameliorative strategies demonstrate restricted efficacy in comprehensively mitigating heat stress-associated pathophysiological insults [7]. These approaches often fail to maintain productivity when ambient temperatures exceed 32 °C, where physiological disturbances become severe and conventional interventions reach their limits [8]. Moreover, their deployment necessitates considerable capital expenditure in infrastructure and technology, thereby presenting formidable economic barriers for smallholder or resource-limited poultry enterprises. Specifically, strategies that enhance hepatic antioxidant defense, minimize inflammation, and stabilize metabolic pathways hold promise for mitigating heat stress impacts on laying hens [9]. While pharmacological antioxidants such as vitamin E and N-acetylcysteine have been explored, their limited bioavailability and weak membrane penetration reduce their efficacy, with studies showing that supplementing vitamin E at 250 mg/kg diet reduces oxidative stress markers by only 20~30% under heat stress conditions, which is insufficient to prevent hepatic lipid peroxidation and mitochondrial dysfunction [10,11]. Additionally, synthetic additives face growing regulatory restrictions and consumer preference for natural alternatives. This highlights a significant research gap: the development of natural, hepatoprotective compounds capable of addressing the multifaceted challenges of heat stress in poultry. This necessitates novel, natural hepatoprotectants targeting redox-sensitive heat stress pathways.

Silibinin, a standardized flavonolignan complex derived from milk thistle seeds, has attracted increasing attention owing to its potent hepatoprotective, anti-inflammatory, and lipid-regulating properties [12]. Silibinin effectively neutralizes reactive oxygen species (ROS), including hydroxyl and peroxyl radicals, and concurrently augments endogenous antioxidant defenses by upregulating SOD, CAT, and GSH-Px [13]. These antioxidant mechanisms are crucial for mitigating oxidative stress-induced liver damage, particularly in conditions such as NAFLD and drug-induced hepatotoxicity [14]. Beyond its antioxidant capacity, silibinin exerts profound regulatory effects on hepatic lipid metabolism. It suppresses de novo lipogenesis by downregulating sterol regulatory element-binding protein 1c (SREBP-1c) and its downstream targets, including fatty acid synthase (FAS) and acetyl-CoA carboxylase (ACC), thereby reducing triglyceride (TG) accumulation [15]. Silibinin additionally alleviates hepatic steatosis by enhancing the secretion of very-low-density lipoprotein (VLDL) through the upregulation of microsomal triglyceride transfer protein (MTTP) expression [15,16]. Importantly, silibinin exhibits an excellent safety margin, and its dual antioxidant and lipid-regulating actions position it as a promising candidate. This could enhance resilience in smallholder systems, supporting UN Sustainable Development Goals amid rising temperatures.

In this study, we aimed to delineate the mechanisms by which dietary silibinin alleviates heat stress-induced hepatic lipid metabolism disorder in laying hens. We attempted to establish a causal relationship between SIL-mediated steady-state improvement in laying hens by integrating performance parameters, egg quality index, plasma metabolomics, and liver transcriptomics. These findings will not only expand our understanding of liver protection mechanisms based on natural extracts in poultry, but also provide a practical, cost-effective nutritional strategy to enhance poultry resilience to face severe climate change.

## 2. Results

### 2.1. Silibinin Enhances Laying Rate and Egg Quality of Laying Hens Under Heat Stress

**Figure 1:**
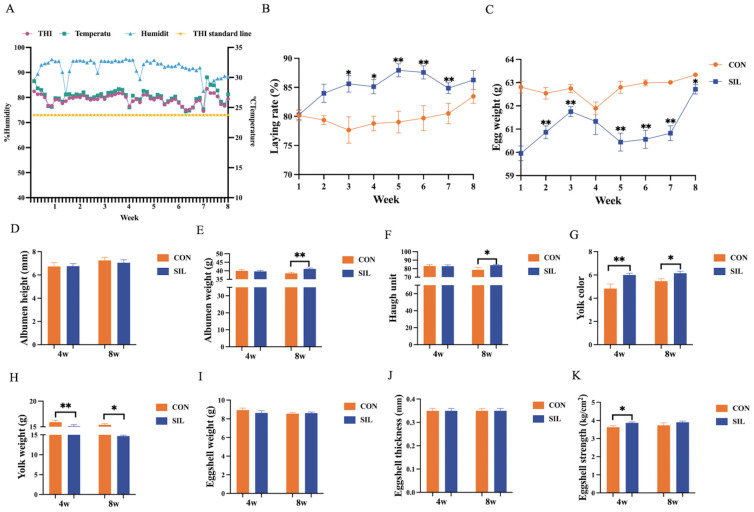
Effect of silibinin on laying rate and egg quality of laying hens under heat stress. (**A**) Temperature and humidity index curve. (**B**) Effects of silibinin on laying rate and (**C**) egg weight, (**D**) albumen height, (**E**) albumen weight, (**F**) Haugh unit, (**G**) yolk color, (**H**) yolk weight, (**I**) eggshell weight, (**J**) eggshell thickness, and (**K**) eggshell strength. Data are presented as mean ± SEM, (**B**,**C**), *n* = 6; (**D**–**K**), *n* = 24. Data in (**B**,**C**) were analyzed by Two-way Repeated Measures ANOVA followed by Sidak’s multiple comparisons test. Data in (**D**–**K**) were analyzed by independent Student’s *t*-test. Data are presented as mean ± SEM. * *p* < 0.05, ** *p* < 0.01. CON: a basal diet; SIL: a basal diet supplemented with 100 mg/kg silibinin.

Throughout the experimental period, the temperature–humidity index consistently exceeded 73, confirming the establishment of a robust heat stress model (Figure 1A). Dietary silibinin significantly mitigated HS-induced losses, elevating laying rate (Figure 1B) and gradually improving the weight of eggs relative to the unsupplemented control. Part of the egg quality has a time effect, with a significant increase in albumen height and Haugh units in the last four weeks (Figure 1E,F). Egg yolk color significantly increases with the feeding of silibinin (Figure 1G), and the eggshell strength of the SIL group significantly increases in the first four weeks (Figure 1K), while egg yolk weight shows a significant decrease during the feeding stage (Figure 1H).

### 2.2. Silibinin Alleviates Hepatic Steatosis in Laying Hens Under Heat Stress

**Figure 2:**
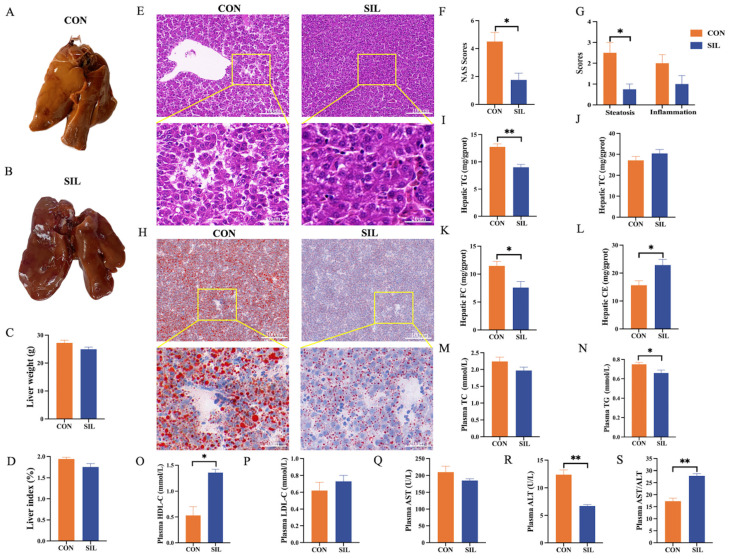
Effect of silibinin on liver lipid metabolism in laying hens under heat stress. (**A**,**B**) Gross morphology of the liver. (**C**,**D**) Liver weight and index. (**E**) H&E-stained sections of the liver (scale bars: 100 μm and 20 μm, respectively). (**F**) NAS. (**G**) Histological scores for steatosis and inflammation. (**H**) Oil Red O-stained sections of the liver (scale bars: 100 μm and 20 μm, respectively). (**I**–**L**) Hepatic lipid levels. (**M**–**P**) Plasma lipid levels. (**Q**–**S**) Liver injury indicators. Statistical differences were determined by independent two-tailed Student’s *t*-test. Data are presented as mean ± SEM, *n* = 6. * *p* < 0.05, ** *p* < 0.01.

Livers from control hens exhibited a yellowish, greasy surface indicative of severe steatosis (Figure 2A), whereas those from the silibinin group retained a normal bright-red appearance (Figure 2B). Histopathological assessment via HE staining revealed markedly fewer cytoplasmic vacuoles and a concomitant reduction in NAS (Figure 2E–G), confirming attenuated hepatic lipid accumulation. Oil Red O staining corroborated a pronounced decrease in lipid droplet density (Figure 2H). Hepatic TG and FC concentrations were significantly diminished (Figure 2I–K), whereas CE content was elevated (Figure 2L). Plasma TG and ALT activities were concurrently decreased (Figure 2N–O), while HDL-C levels and the aspartate aminotransferase/alanine aminotransferase ratio (AST/ALT) were elevated. Collectively, dietary silibinin effectively mitigated heat stress-induced hepatic steatosis and ameliorated associated hepatic injury in laying hens.

### 2.3. Silibinin Enhances the Antioxidant Capacity of Laying Hens

**Figure 3:**
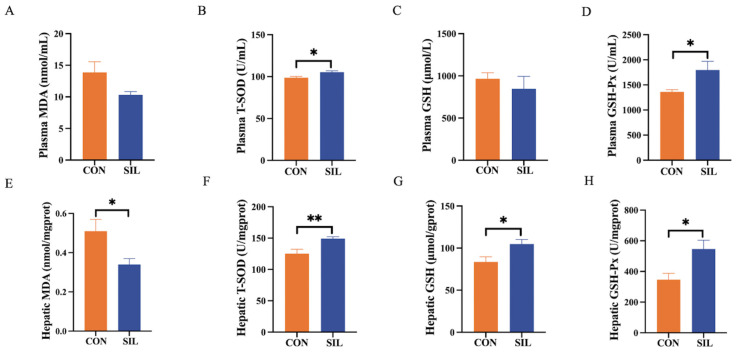
Effects of silibinin on antioxidant capacity in heat-stressed laying hens. (**A**) Effect of silibinin on plasma MDA and (**B**) plasma T-SOD, (**C**) plasma GSH, and (**D**) plasma GSH-Px. (**E**) Effect of silibinin on hepatic MDA and (**F**) hepatic T-SOD, (**G**) hepatic GSH, and (**H**) hepatic GSH-Px. Statistical differences were determined by independent two-tailed Student’s *t*-test. Data are presented as mean ± SEM, *n* = 6. * *p* < 0.05, ** *p* < 0.01. Abbreviations: MDA, malondialdehyde; T-SOD, total superoxide dismutase; GSH, glutathione; GSH-Px, glutathione peroxidase.

Eight weeks of dietary silibinin elevated both plasma and hepatic T-SOD and GSH-Px activities while boosting hepatic GSH reserves (Figure 3B,D,F–H); this antioxidant reinforcement coincided with a marked decline in hepatic MDA (Figure 3E), indicating reduced oxidative injury and improved liver health in heat-stressed laying hens.

### 2.4. Silibinin Alters Plasma Metabolites

**Figure 4:**
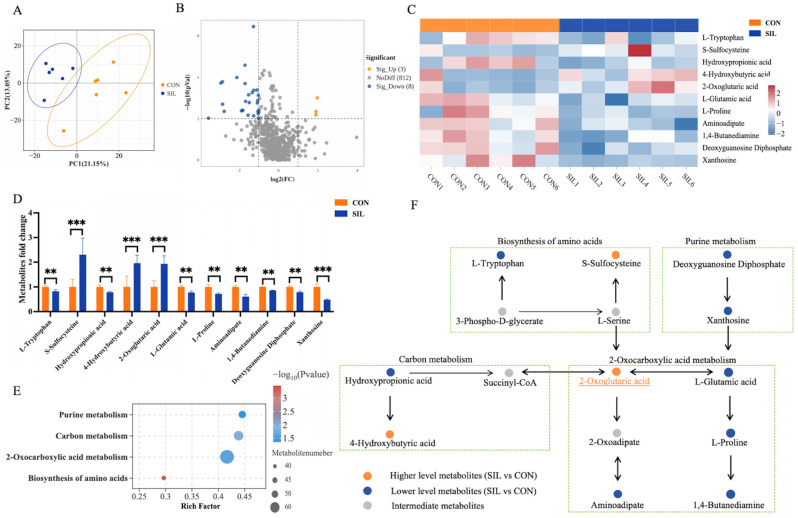
Effects of silibinin on plasma metabolites in heat-stressed laying hens. (**A**) Principle component analysis score plot of two group samples. (**B**) Volcano map of differentially regulated metabolites. (**C**) Differential metabolite heatmap. (**D**) Differential metabolite fold change bar graph. (**E**) Enriched metabolic pathways related network diagram. (**F**) Key metabolite synthesis process. Statistical differences were determined by independent two-tailed Student’s *t*-test. Data are presented as mean ± SEM, *n* = 6. ** *p* < 0.01, *** *p* < 0.001. Significantly differential metabolites were identified based on variable importance in projection (VIP) > 1, fold change (FC) > 2 (or <0.5), and *p* < 0.05.

Principal component analysis (PCA) exhibited a marked distinction between the two groups (Figure 4A). Significantly different metabolites were identified using a fold change (FC) threshold of either greater than 2 or less than 0.5, with a significance level of *p* < 0.01, We identified eleven differential metabolites, three of which were upregulated, S-sulfocysteine, 4-hydroxybutyric acid, and 2-oxoglutaric acid, and eight of which were downregulated: L-tryptophan, hydroxypropionic acid, L-glutamic acid, L-proline, aminoadipate, 1,4-butanediamine, deoxyguanosine diphosphate, and xanthosine (Figure 4B–D). Pathway enrichment indicated that these metabolites are mainly involved in 2-oxocarboxylic acid metabolism, purine metabolism, carbon metabolism, and amino acid metabolism (Figure 4E), with 2-oxoglutaric acid serving as a central hub and 2-oxocarboxylic acid metabolism as the most affected pathway (Figure 4F).

### 2.5. Silibinin Regulates Liver Lipid Metabolism

**Figure 5:**
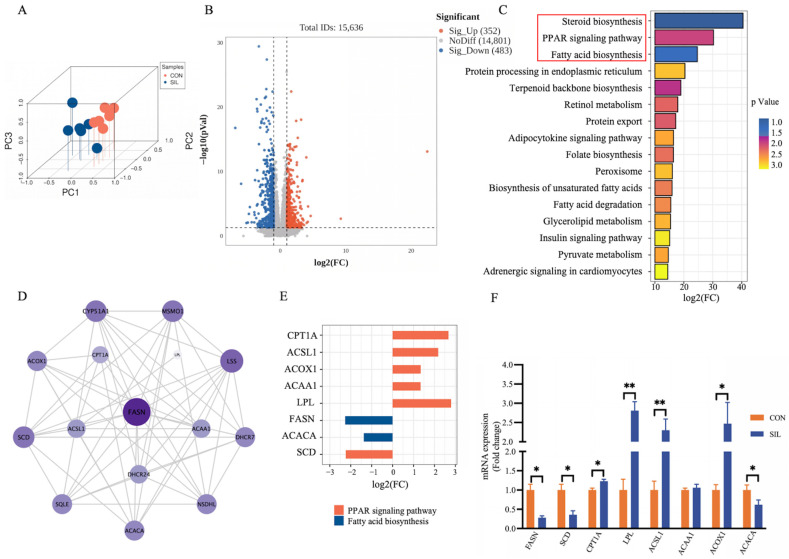
Hepatic transcriptomics. (**A**) 3D principal component analysis (PCA) score plot of two group samples. (**B**) Volcano plot of differentially expressed genes. (**C**) The top 16 significantly enriched KEGG pathways. (**D**) Protein–protein interaction (PPI) network of the differentially expressed genes in top three pathways. (**E**) Bar chart of key gene fold changes. (**F**) qPCR to verify the expression of differential genes. Statistical differences were determined by independent two-tailed Student’s *t*-test. Data are presented as mean ± SEM, *n* = 6. * *p* < 0.05, ** *p* < 0.01. Differentially expressed genes (DEGs) were defined by |log2(FC)| > 1 and FDR-adjusted *p* < 0.05.

The three-dimensional PCA plot showed a clear separation between the two liver groups (Figure 5A). The volcano plot, applying *p* < 0.05 and |FC| > 2, uncovered 352 upregulated and 483 downregulated genes (Figure 5B). Functional enrichment highlighted steroid biosynthesis, PPAR signaling, and fatty acid biosynthesis as the top three affected pathways (Figure 5C). Protein–protein interaction analysis positioned FASN as the central hub, most tightly linked to PPAR-related genes (Figure 5D). Six upregulated genes mapped to PPAR signaling, while two downregulated genes belonged to fatty acid biosynthesis (Figure 5E). Quantitative PCR analysis corroborated the expression profiles of seven out of eight selected DEGs, confirming the reliability of our transcriptomic data. The exception was ACAA1, which exhibited an upward trend consistent with RNA-seq but failed to reach statistical significance (*p* > 0.05). This suggests that while ACAA1 may participate in the response, it is likely not a dominant driver of the observed lipid catabolism compared to upregulated targets like CPT1A and ACSL1 (Figure 5F). These data indicate that silibinin activates PPAR signaling and represses fatty acid biosynthesis in heat-stressed layers.

### 2.6. Silibinin Suppresses Hepatic De Novo Lipogenesis and Promotes Fatty Acid β-Oxidation

**Figure 6:**
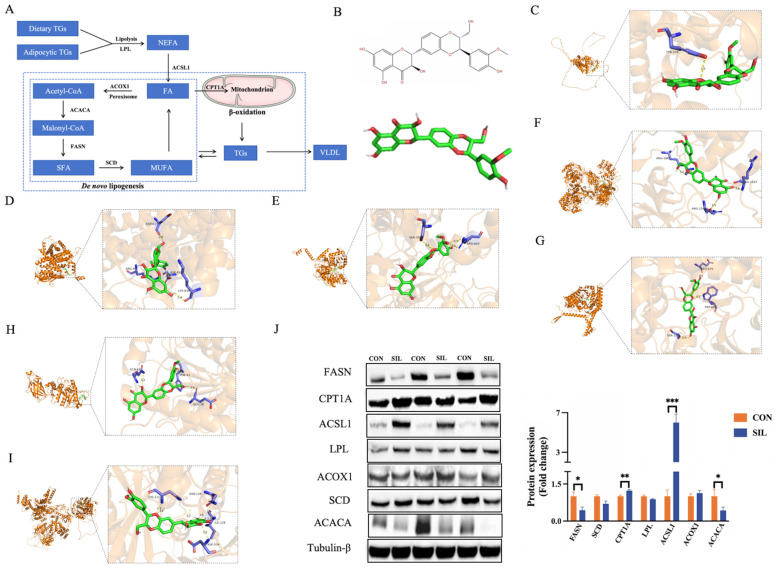
Analysis and verification of critical regulatory targets. (**A**) Schematic diagram of the regulatory mechanism of silibinin on liver lipid metabolism. (**B**) 2D and 3D structural diagrams of silibinin. (**C**–**I**) Molecular docking diagram of silibinin and core targets; yellow dashed lines indicate predicted hydrogen bonds interacting with key amino acid residues. (**J**) Western blot analysis of the differentially expressed proteins. Statistical differences were determined by independent two-tailed Student’s *t*-test. Data are presented as mean ± SEM, *n* = 6. * *p* < 0.05, ** *p* < 0.01, *** *p* < 0.001.

A concise diagram summarizes the lipid metabolism regulatory network involved in the key genes regulated by silibinin (Figure 6A). 2D and 3D structural diagrams of silibinin are provided (Figure 6B), followed by snapshots of its docked results within the catalytic pockets of SCD, ACOX1, ACSL1, FASN, CPT1A, LPL and ACACA (Figure 6C–I). Among these interactions, CPT1A produced the lowest binding energy (Table 1), indicating that the predicted complex with silibinin was the most stable. These simulations suggest a high degree of structural compatibility and favorable binding energy between silibinin and its putative lipid metabolism targets. Among the core targets, Western blot confirmed that ACACA, FASN, CPT1A, and ACSL1 changed in line with qPCR data, underscoring their pivotal role (Figure 6J). These dual effects converge to suppress de novo lipogenesis and accelerate fatty acid β-oxidation, thereby restoring hepatic lipid balance.

### 2.7. Correlation Analysis

**Figure 7:**
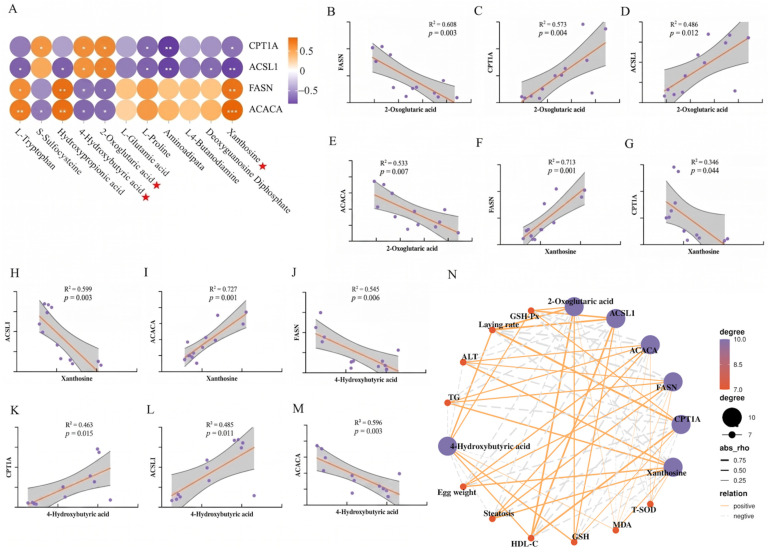
Correlation and nonlinear regression analysis between key metabolites and key proteins. (**A**) Correlation heatmap between differential metabolites and key proteins. (**B**) Nonlinear regression analysis 2-oxoglutaric acid and FASN, and (**C**) CPT1A, (**D**) ACSL1, and (**E**) ACACA. (**F**) Nonlinear regression analysis xanthosine and FASN, and (**G**) CPT1A, (**H**) ACSL1, and (**I**) ACACA. (**J**) Nonlinear regression analysis 4-Hydroxybutyric acid and FASN, and (**K**) CPT1A, (**L**) ACSL1, and (**M**) ACACA. (**N**) Correlation between regulatory targets of silibinin. Statistical differences were determined by independent two-tailed Student’s *t*-test. Data are presented as mean ± SEM, *n* = 6. * *p* < 0.05, ** *p* < 0.01, *** *p* < 0.001.

**Figure 8:**
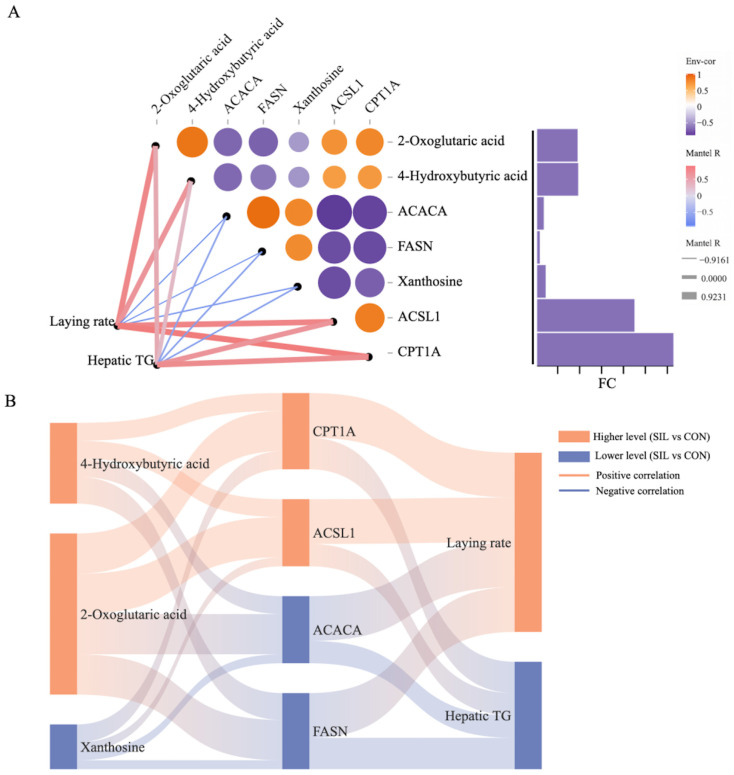
Correlation analysis. (**A**) Heatmap of correlation between key differential proteins, key differential metabolites, and apparent indicators. (**B**) Sankey diagram of key differential proteins, key differential metabolites, and apparent indicators. *n* = 6.

Correlation heatmaps revealed tight links between key metabolites and proteins: 4-hydroxybutyric acid, 2-oxoglutaric acid, and xanthosine emerged as the most influential (Figure 7A). Nonlinear regression analysis revealed a strong correlation between 2-oxoglutaric acid and FASN, with a high model fit (Figure 7B). Similarly, xanthine, 4-hydroxybutyric acid, and ACACA showed strong correlations with high model fit (Figure 7I,M). Liver antioxidant indicators T-SOD, GSH, and GSH Px have a strong positive correlation with 4-hydroxybutyric acid, 2-oxoglutaric acid, CPT1A, and ACSL1, but a strong negative correlation with xanthine, ACACA, and FASN; egg weight and plasma HDL-C also have the same correlation, while MDA, ALT, and liver steatosis show the opposite trend (Figure 7N). Laying rate is significantly positively correlated with key metabolites 4-hydroxybutyric acid and 2-oxoglutaric acid, significantly negatively correlated with xanthine, significantly positively correlated with core targets CPT1A and ACSL1, and significantly negatively correlated with ACACA and FASN (Figure 8A,B). Integrative pathway analysis revealed that elevated plasma levels of 4-hydroxybutyric acid and 2-oxoglutaric acid were associated with enhanced fatty acid β-oxidation and concurrent suppression of lipogenesis, whereas xanthosine exhibited an opposing regulatory influence on these metabolic pathways. Hepatic TG is significantly negatively correlated with key metabolites 4-hydroxybutyric acid and 2-oxoglutaric acid, and significantly positively correlated with xanthine, core targets CPT1A and ACSL1, and ACACA and FASN (Figure 8A,B).

## 3. Discussion

This study provides a comprehensive exploration of the effects of dietary silibinin on heat-stressed laying hens through an integrative multi-omics approach. The findings demonstrate that silibinin at 100 mg/kg can significantly counteract the detrimental impacts of heat stress on liver health and egg production performance. By employing a multi-faceted research strategy that combines transcriptomic, metabolomic, and functional analyses, we have unveiled the molecular mechanisms through which silibinin ameliorates hepatic lipid metabolism and oxidative stress. This work advances our understanding of the role of natural bioactive compounds in mitigating the adverse effects of climatic stressors on livestock productivity. The results not only validate silibinin as a promising feed additive but also offer valuable insights for the development of targeted nutritional interventions in the poultry industry. The potential for silibinin to serve as a cornerstone in sustainable and resilient poultry production strategies amid escalating global temperatures underscores the significance of this research. Future studies could further explore the long-term effects of silibinin supplementation, its interactions with other nutrients, and its applicability across different poultry species and production systems.

Heat stress exerts multifaceted detrimental effects on laying hens, significantly impairing their production performance, egg quality, and physiological homeostasis. Chronic heat stress has been consistently shown to reduce egg production rate, egg weight, and eggshell quality, primarily due to decreased feed intake and disrupted nutrient metabolism [17,18]. Notably, silibinin linearly improved laying rate, egg weight, and feed conversion ratio (FCR) at doses of 0.02~0.06% [19]. From a practical standpoint, the observed dose-dependent effects of silibinin (0.02~0.06%) on laying performance and egg quality highlight its potential as an economically viable feed additive for mitigating HS-induced losses in poultry production. However, further studies are needed to evaluate its stability under extreme environmental conditions and its efficacy in other livestock species. The decline in eggshell strength and thickness under heat stress is attributed to metabolic alkalosis caused by panting, which reduces blood bicarbonate availability for shell mineralization [20]. In this study, egg quality such as Haugh unit, albumen height, and yolk color were enhanced, due to the antioxidant properties of silibinin; it stabilizes the homeostasis of laying hens, reduces inflammation, and improves metabolism in the body [21,22].

Additionally, heat stress disrupts plasma biochemical parameters, including elevated creatinine and reduced albumin levels, indicating renal dysfunction [20], as well as increased triglycerides and cholesterol due to altered lipid metabolism [22]. Liver health is particularly compromised, with heat stress inducing oxidative damage, mitochondrial dysfunction, and hepatic lipid accumulation, leading to fatty liver syndrome [18,23]. However, in this study, silibinin reversed this situation: plasma biochemical analyses revealed reduced cholesterol and TG levels, alongside lowered NAS and ALT activities, indicating improved lipid metabolism and hepatoprotection.

Heat stress induces oxidative damage and lipid peroxidation, leading to elevated serum ALT, AST, and cholesterol levels; however, silibinin reversed this situation in our work, and counteracted these effects by reducing MDA and enhancing SOD and GSH-Px activities, thereby preserving hepatic function [24]. And the key metabolites in the plasma metabolome reshaped by silibinin are closely related to antioxidant capacity. A pivotal observation herein is the silibinin-elicited augmentation of 2-oxoglutarate (α-KG), a central TCA cycle intermediary pivotal to mitochondrial bioenergetics. A-KG enhances antioxidant defenses by upregulating GSH levels and reducing reactive oxygen species (ROS) accumulation [25,26]. In this study, these effects are further supported by silibinin-induced activation of antioxidant enzymes such as SOD and GSH-Px, suggesting a dual mechanism for mitigating oxidative stress. A-KG enhances mitochondrial fatty acid oxidation through two distinct mechanisms. First, α-KG activates AMP-activated protein kinase (AMPK) in hepatocytes, initiating downstream signaling that increases the phosphorylation of ACC. This phosphorylation inhibits ACC activity, reducing malonyl-CoA levels and subsequently relieving the inhibition of carnitine palmitoyltransferase 1 (CPT1) [27,28]. Second, α-KG serves as a substrate for the mitochondrial enzyme glutamate dehydrogenase (GDH), generating NADH that fuels the electron transport chain and improves oxidative phosphorylation efficiency [29]. This dual action creates a metabolic shift favoring lipid catabolism over storage. Silibinin enhances α-KG levels primarily by stimulating the tricarboxylic acid (TCA) cycle and mitochondrial metabolism. 4-hydroxybutyric (GHB) inhibits lipid synthesis and CO_2_ production in mitochondria-containing tissues, including liver; this inhibition likely stems from GHB’s interference with acetyl-CoA metabolism, a critical substrate for fatty acid synthesis, or its indirect suppression of mitochondrial respiration [30]. Silibinin’s antioxidant properties reduce oxidative degradation of GHB, thereby stabilizing its plasma concentration [31,32]. Xanthosine (XTS), a purine nucleoside, modulates lipid metabolism through PPAR signaling, enhancing fatty acid uptake and utilization [33]. The mechanistic interplay between xanthosine’s antioxidant and lipid-modulating properties appears centered on its ability to regulate mitochondrial function by maintaining mitochondrial membrane potential and enhancing oxidative phosphorylation capacity; xanthosine preserves cellular energy homeostasis while minimizing ROS generation [34]. This mitochondrial stabilization, potentially linked with GPX4 upregulation [35], contributes to reducing ferroptosis-induced hepatocyte damage, although further validation is required. However, the reduction in plasma xanthosine reflects enhanced hepatic uptake and intracellular metabolism of xanthosine, leading to its accelerated conversion into bioactive derivatives in the liver. Xanthosine is metabolized by purine nucleoside phosphorylase (PNP) to generate xanthine, which is further processed into uric acid—a potent antioxidant at physiological levels [36,37]. Silibinin enhances XTS levels by inhibiting xanthine oxidase (XO), an enzyme that degrades XTS into xanthine and uric acid [37].

Unlike previous studies primarily focusing on silibinin’s antioxidant properties, our research reveals a novel, multi-target mechanism in which silibinin simultaneously modulates key lipogenic and oxidative pathways (ACACA, FASN, CPT1A, and ACSL1). The integration of plasma metabolomics and liver transcriptomics provides unprecedented insights into the dynamic interactions between lipid metabolism and antioxidant defenses. At the molecular level, we observed that silibinin significantly downregulated ACACA expression, consistent with the findings of Xie et al. [37]. This inhibition reduces malonyl-CoA production, which serves dual roles: (1) as a substrate for fatty acid elongation and (2) as an allosteric inhibitor of CPT1A [38]. Our findings reveal that silibinin downregulates key lipogenic genes, including ACACA and FASN, thereby reducing malonyl-CoA production and limiting long-chain fatty acid synthesis. Additionally, the upregulation of CPT1A and ACSL1 promotes mitochondrial fatty acid oxidation. Molecular docking results further confirm that silibinin binds to the regulatory domains of these enzymes, suggesting a direct modulatory effect on lipid metabolic pathways [39]. These effects are mediated through silibinin’s inhibition of SREBP-1 nuclear translocation and transcriptional activity [40]. Our data revealed that silibinin treatment markedly upregulated CPT1A expression, facilitating mitochondrial fatty acid β-oxidation. This effect appears to be mediated through PPARα activation, as evidenced by increased PPARα binding to the CPT1A promoter region [41]. Furthermore, we identified ACSL1 as another key target of silibinin, with treatment significantly enhancing ACSL1 protein levels. This finding corroborates the work of Zhou et al. [42], who demonstrated that ACSL1 activation promotes fatty acid channeling toward oxidative pathways rather than esterification. The coordinated regulation of these four key targets (ACACA, FASN, CPT1A, and ACSL1) by silibinin creates a metabolic shift in hepatocytes from lipid accumulation to lipid utilization. This multi-target mechanism explains the compound’s efficacy in ameliorating hepatic steatosis in NAFLD models [43]. It is important to note that the molecular docking results presented here are predictive in nature. While the low binding energies (−9.7 kcal/mol for CPT1A) provide a theoretical structural rationale for Silibinin’s regulatory effects, they do not empirically prove direct in vivo target engagement. These in silico findings serve as suggestive evidence that warrants future validation through biophysical assays such as Surface Plasmon Resonance (SPR) or enzyme activity inhibition tests. While our integrative multi-omics approach provides a holistic view of silibinin’s role under heat stress, we emphasize that the identified protein–metabolite–phenotype networks are fundamentally associative. These findings constitute a molecular roadmap that points toward candidates for future validation via direct enzymatic assays or tissue-specific genetic interventions. The minor discrepancy between RNA-seq and qPCR for ACAA1 does not fundamentally alter our pathway-level conclusions. In complex biological systems like the avian liver, redundant enzymatic activities often compensate for individual gene fluctuations. The high degree of concordance among other rate-limiting enzymes in the fatty acid β-oxidation and de novo lipogenesis pathways confirms that silibinin exerts a systemic, rather than isolated, regulatory effect on hepatic lipid homeostasis. The concurrent repression of lipogenesis and augmentation of β-oxidative capacity positions silibinin in a distinctive therapeutic niche compared with extant interventions that conventionally address only a singular facet. To further elucidate the hepatoprotective mechanism of silibinin, it is essential to integrate our metabolomic and transcriptomic findings, which collectively highlight a highly coordinated systemic metabolic shift from hepatic lipid storage to utilization under heat stress. Our metabolomic analysis revealed a significant silibinin-induced elevation in plasma 2-oxoglutaric acid (α-KG) and 4-hydroxybutyric acid, alongside a reduction in xanthosine. Crucially, this systemic metabolic milieu reshaped by silymarin synergizes directly with the local hepatic transcriptional reprogramming. For instance, α-KG, a critical TCA cycle intermediate, not only enhances mitochondrial bioenergetics but also serves as a potent signaling molecule that promotes lipid catabolism. This systemic metabolic state aligns perfectly with our transcriptomic data, which demonstrated the significant upregulation of hepatic CPT1A and ACSL1, the rate-limiting enzymes for fatty acid β-oxidation. Conversely, the reduction in purine metabolites like xanthosine corresponds strongly with the transcriptional suppression of de novo lipogenesis pathways, evidenced by the marked downregulation of ACACA and FASN. The shifts in circulating metabolites, particularly the increased availability of α-KG, are highly congruent with the observed hepatic transcriptional remodeling, potentially contributing to the relief of allosteric inhibition on CPT1A. This integration relieves the allosteric inhibition on CPT1A and accelerates mitochondrial fatty acid oxidation, thereby mechanistically resolving heat stress-driven hepatic steatosis. A critical methodological consideration in this study is the formulation of the basal diet. It is pertinent to discuss the rationale behind the formulation of the basal diet used in this study (ME = 2.61 Mcal/kg). While this energy level is lower than typical commercial recommendations for thermoneutral conditions, it was a deliberate methodological choice. Conventional strategies to combat heat stress-induced energy deficits often involve supplementing dietary fat to increase energy density without elevating the Heat Increment of Feeding (HIF) [44]. However, the influx of exogenous dietary lipids would serve as a major confounding factor when evaluating hepatic de novo lipogenesis and endogenous lipid accumulation. By utilizing a moderate ME level without excessive fat supplementation, we established a strict metabolic decompression baseline. This precise formulation prevented exogenous lipid-induced artifacts, ensuring that the severe hepatic steatosis observed in the control group was purely driven by heat stress, and allowing for a rigorous, unconfounded evaluation of silibinin’s regulatory mechanisms on lipid metabolism targets. Importantly, the adoption of this moderate ME level did not precipitate energy starvation, which could theoretically confound lipid metabolism by forcing compensatory hepatic lipid mobilization. Phenotypic data confirmed that daily energy intake (339 kcal/bird, derived from 130 g/day feed allocation) remained sufficient to support robust laying rates and egg weights in both groups. Furthermore, the presence of severe, macrovesicular hepatic steatosis in the control group decisively refutes any hypothesis of starvation-induced lipid depletion. Consequently, the mitigation of hepatic lipid accumulation observed in the treatment group can be confidently attributed to silibinin’s specific pharmacological modulation of lipogenic and oxidative pathways, rather than a generalized nutritional deficit.

Despite the comprehensive multi-omics insights provided, the present study has certain experimental limitations that warrant consideration. Primarily, the experimental design lacked a thermoneutral (TN) control group. Our primary objective was to evaluate the hepatoprotective and restorative efficacy of silymarin under a continuous, clinically relevant heat stress model (THI > 73) that closely mimics commercial summer poultry production conditions. However, we acknowledge that without a parallel TN baseline, it is difficult to fully quantify the absolute magnitude of the physiological and metabolic deviations induced exclusively by heat stress, as well as the exact proportional extent to which silymarin restores these parameters to pre-stress levels. Furthermore, distinguishing between targeted stress-alleviation and general basal metabolic improvement requires comparative baselines as emphasized in recent poultry thermal biology reviews. Future research should incorporate TN and TN + silibinin groups to systematically decouple the molecular responses specific to silibinin from its stress-mitigating properties, thereby providing a more holistic assessment of its therapeutic magnitude. As emphasized in recent methodological reviews of poultry thermal biology, evaluating the absolute efficacy of nutritional interventions ideally requires comparative baselines to distinguish between targeted stress-alleviation and general basal metabolic improvements [2,7]. Therefore, future studies should incorporate both TN and TN-plus-silymarin control groups to systematically decouple the specific molecular responses to silymarin from its stress-mitigating properties, thereby providing a more holistic evaluation of its therapeutic magnitude. Another limitation of the current experimental design is the reliance on a single supplementation level of silibinin (100 mg/kg). Although this specific concentration was strategically selected based on recent evidence demonstrating its potent efficacy in modulating hepatic lipid metabolism and alleviating oxidative stress in poultry [15], utilizing a single dose restricts our capacity to fully characterize potential dose-dependent responses. The recent literature indicates that varying inclusion rates of silymarin can exert differential, and sometimes linear, improvements on laying performance, liver health, and lipid profiles [19]. Consequently, without a graded dose–response evaluation, identifying the minimal effective concentration, the saturation point of its bioactivity, or the optimal economic inclusion threshold remains challenging. Therefore, future comprehensive studies implementing a multi-dose gradient design (e.g., ranging from 50 to 200 mg/kg) are warranted to systematically delineate the dose–response kinetics and establish precision nutritional guidelines for silibinin application in heat-stressed livestock.

## 4. Materials and Methods

### 4.1. Breeding and Management of Animals

A total of 252 healthy Hy-Line Brown laying hens (43 weeks of age), exhibiting uniform egg production during the peak laying period, were randomly assigned to two experimental groups. Each group comprised six replicate pens, with 21 hens per pen. Then a two-week pre-experiment was conducted, and the cage position was adjusted according to the weekly egg laying rate to ensure that there was no difference in the egg laying rate within and between groups before the formal test. A controlled heat stress (HS) regimen was applied daily from 07:00 to 15:00, maintaining ambient temperature at 32 ± 1 °C and relative humidity at 65 ± 5%, yielding a temperature–humidity index that clearly exceeds the established thermoneutral threshold (THI = 73) for laying hens. In order to study the effect of silibinin on the late laying hens under heat stress at the same nutritional level, the control group was fed a basal diet, and the experimental groups received the same basal diet supplemented with 100 mg/kg of silibinin (a highly purified monomeric compound, 98% purity) sourced from Dong Feng Bio-Technique Co., Ltd, Xi’an, China. According to the manufacturer’s specifications, the remaining 2% non-silibinin fraction consists exclusively of trace moisture and structurally related stereoisomers (isosilybin), entirely devoid of macromolecular immunogenic contaminants (such as endotoxins) that could otherwise confound inflammatory or metabolic evaluations. The 10-week study comprised a 2-week acclimation phase followed by an 8-week experimental phase. Throughout, birds were exposed to a 16L:8D photoperiod and provided water ad libitum; daily feed allocation was restricted to 130 g/bird to minimize feed refusal artifacts. Daily records were maintained for egg production rates (%) and egg weight (g), with egg quality assessments conducted at 4-week intervals. The animal experiments were approved by the Institutional Animal Care and Use Committee of Nanjing Agricultural University (Approval Number: NJAULLSC2024062). Details regarding the composition and nutritional specifications of the basal diet are presented in Tables S1 and S2. Crude protein was determined by the Kjeldahl method (AOAC 984.13), crude fat by Soxhlet extraction (AOAC 920.39), and calcium and total phosphorus by ICP-OES. The apparent metabolizable energy (AME) was calculated based on the analyzed chemical composition using validated avian energy prediction equations.

### 4.2. Egg Quality

Egg quality indices were assessed at 4-week intervals using a representative subsample of 30 eggs per group. From each of the 6 replicates per group, we randomly selected 5 eggs, yielding a total of 30 eggs per treatment. Among these, 24 eggs (4 per replicate) were analyzed for internal quality, while 6 eggs (1 per replicate) were reserved as standbys to account for potential breakage during handling. Measured variables included shell mass (g) and its percentage of total egg weight, yolk weight (g) and yolk ratio (%), albumen weight (g) and albumen ratio (%), albumen height (mm), yolk color score, Haugh unit, eggshell strength (kg/cm^2^), and eggshell thickness (mm). Fracture resistance was quantified with an impact tester (Model WW-2A, Soil Instrument Factory, Nanjing, China) operated at a constant loading rate until shell failure. Shell thickness was measured at three equidistant points, using a precision micrometer (0.01 mm resolution); the mean of the three readings, excluding the inner and outer shell membranes, was reported. Remaining traits were determined non-destructively by near-infrared analysis with a multifunctional egg analyzer (EMT-5200, Robotmation Co., Tokyo, Japan) calibrated daily against reference standards. All measurements were performed in triplicate per egg under controlled ambient conditions (25 °C, 60 ± 5% relative humidity).

### 4.3. Sample Collection

After analyzing laying rate and egg quality, we picked ten hens from each group. At the end of the experiment, one hen was randomly selected from each of the three cages within every replicate, yielding an initial pool of 18 hens (3 hens/replicate × 6 replicates). To ensure physiological uniformity and eliminate body weight outliers, these 18 hens were weighed, and 10 hens whose body weights were closest to the average weight of the entire flock (within a 5% margin) were finally selected for sampling (yielding *n* = 10 per treatment). This targeted selection strategy, as opposed to complete randomization, was deliberately employed to minimize basal metabolic noise introduced by extreme phenotypes (natural emaciation or obesity). By reducing within-group biological variance, this procedure ensured maximum biological representativeness of the typical flock response and improved the signal-to-noise ratio for subsequent high-dimensional multi-omics analyses. This procedure ensured maximum biological representativeness while preventing cage-effect bias. Blood was obtained by brachial venipuncture, transferred to collecting tubes (with heparin sodium), and immediately centrifuged (3000× *g*, 10 min, 4 °C); plasma samples were placed on dry ice, and then stored in a −20 °C refrigerator. Segments of liver were excised and rinsed in ice-cold 0.9% saline solution. Tissue samples were then divided into two portions: one was fixed in 4% paraformaldehyde at 4 °C for 24 h for histological analysis, and the other was rapidly frozen in liquid nitrogen and stored at −80 °C for subsequent molecular and biochemical assays.

### 4.4. Plasma Biochemistry and Metabolomics

Plasma metabolic profiles, including total cholesterol (TC), triglyceride (TG), high-density lipoprotein-cholesterol (HDL-C), low-density lipoprotein-cholesterol (LDL-C), alanine aminotransferase (ALT) and aspartate aminotransferase (AST), were quantified on a Hitachi 7020 automated chemistry analyzer (Tokyo, Japan) using reagents supplied by Sanhe Biotechnology (Nanjing, China). Oxidative status was evaluated by spectrophotometric determination of T-SOD, MDA, GSH-Px, and reduced GSH employing commercial kits (Jiancheng Bioengineering Institute, Nanjing, China).

A non-targeted metabonomics experiment was completed by Biotree Biomedical Technology Co., Ltd. (Shanghai, China). For this procedure, 100 µL of plasma was subjected to extraction with 400 µL of ice-cold methanol/acetonitrile/water (in a 2:2:1 ratio by volume), which was fortified with 2-chloro-L-phenylalanine to facilitate the process. After centrifugation (15,000× *g*, 4 °C, 15 min), supernatants were analyzed by UPLC-HSS-T3 (0.1% formic acid gradient) coupled to a Q-Exactive HF-X Orbitrap operated at 35,000 resolutions in positive/negative modes. Peaks were processed in XCMS, annotated against HMDB (≦5 ppm), and confirmed by MS2 matching. Multivariate and pathway analyses (MetaboAnalyst 6.0) were performed with FDR < 0.05.

### 4.5. Liver Lipid Content and Antioxidant Activity

The hepatosomatic index was derived from aseptically excised liver mass relative to fasted body weight. Hepatic TG, TC, free cholesterol (FC), and cholesterol ester (CE) (CE = TC − FC) were quantified colorimetrically using commercial kits (TG, A110-1-1, Jiancheng Bioengineering Institute, Nanjing, China; TC, E1016-105, FC, E1015-105, Applygen Technologies Inc, Beijing, China). Antioxidant capacity was gauged via T-SOD, GSH-Px, GSH, and MDA contents using validated reagents (Jiancheng Bioengineering Institute, Nanjing, China). All biochemical assays were performed strictly according to the manufacturers’ protocols. For histology, 4% paraformaldehyde-fixed liver was paraffin-embedded and sectioned. Haematoxylin–eosin (HE) and Oil Red O staining were employed to visualize steatosis and lipid deposition; lesions were quantified using the NAFLD activity score (NAS) (steatosis 0–3, inflammation 0–3, ballooning 0–2) by a blinded pathologist. Morphological Assessment (HE): Paraffin-embedded HE staining was utilized to evaluate the overall histological architecture and to quantify the NAFLD activity score (NAS). Chemical Confirmation (Oil Red O): To confirm that these vacuoles were indeed lipid accumulations, we performed Oil Red O staining on frozen sections (cryosections). This technique avoids lipid-dissolving solvents, allowing for the direct visualization of neutral lipids (triglycerides) as bright red droplets. Integration: The high degree of spatial correlation between the cytoplasmic vacuolation in HE sections and the lipid droplets in Oil Red O sections provides robust evidence of hepatic steatosis.

### 4.6. Liver Transcriptomics

Liver specimens were processed for transcriptomic profiling by Shanghai Personal Biotechnology Co., Ltd. (Shanghai, China). Hepatic transcriptome profiling was performed on 30 mg liver fragments cryo-preserved in liquid nitrogen. Concerning biological independence and sample selection for RNA-seq analysis, we utilized six biological replicates (*n* = 6) per treatment group to ensure statistical independence and capture the true biological variance under chronic heat stress. Library Preparation and Sequencing Specifications Platform: Sequencing was performed on the Illumina NovaSeq 6000 platform (Illumina, Inc., San Diego, CA, USA) with a 2 × 150 bp paired-end configuration to ensure high resolution. Depth and Metrics: Our raw data yielded an average of 46.5 million reads per sample. Bioinformatic Pipeline and Parameters: Quality Control: Raw reads were filtered using fastp (version 0.23) to remove adapters and low-quality sequences. Clean reads were aligned to the Gallus gallus GRCg6a reference genome using HISAT2 (version 2.2.1) with default strandedness parameters. Quantification: Feature assignment was executed via feature counts (version 2.0.3). Our unique mapping rate averaged 88.4%, indicating high compatibility between our samples and the reference genome. Differential Expression (DE) Strategy and Thresholds Software: DE analysis was performed using the DESeq2 R package (version 1.30.1), which is optimized for small-sample biological variance. Stringent thresholds: To minimize false positives, we employed Benjamini–Hochberg False Discovery Rate (FDR) correction. Differentially expressed genes (DEGs) were strictly filtered based on an FDR-adjusted *p* < 0.05 and a |log2FoldChange| > 1.

### 4.7. Quantitative Polymerase Chain Reaction

Total RNA was extracted from hepatic tissues using TRIzol reagent (Thermo Fisher Scientific Inc., Waltham, MA, USA, #15596018) and treated with DNase I (Qiagen, Venlo, Netherlands, #79254). RNA concentration and purity were assessed via a NanoDrop 2000 spectrophotometer (A260/A280 > 1.9), and integrity was verified by 1% agarose gel electrophoresis. First-strand cDNA was synthesized from 1 μg of total RNA using the PrimeScript RT Master Mix (Takara Bio USA, Inc., San Jose, CA, USA, #RR036A). Real-time qPCR was performed on a QuantStudio 6 Pro System utilizing PowerUp SYBR Green Master Mix (Applied Biosystems, Thermo Fisher Scientific Inc., Waltham, MA, USA, #A25742). Gene-specific primers (Table S3) were designed via NCBI Primer-BLAST (https://www.ncbi.nlm.nih.gov/tools/primer-blast/, accessed on 7 May 2029) (amplicon size: 90–150 bp; Tm: 60 ± 2 °C). Reactions (10 μL total: 5 μL master mix, 0.5 μM per primer, 20 ng cDNA) underwent UDG activation (50 °C, 2 min), initial denaturation (95 °C, 2 min), and 40 amplification cycles (95 °C for 15 s, 60 °C for 1 min). Amplicon specificity was confirmed by melt curve analysis (yielding a single peak) and representative gel electrophoresis. No-template controls (NTCs) and inter-plate calibrators were included in all runs. Primer amplification efficiencies, determined via 5-point serial dilutions, ranged from 90% to 110% (R^2^ > 0.99). Relative mRNA expression was calculated using the 2^−ΔΔCt^ method, normalized to the internal reference gene β-actin. All assays were performed with six biological replicates and triplicate technical repeats, maintaining intra- and inter-assay coefficients of variation below 5% and 12%, respectively.

### 4.8. Molecular Docking

Two-dimensional and three-dimensional structures of silibinin were retrieved from PubChem and imported into ChemBio3D for energy minimization; the resulting conformers were saved in SDF format. The crystal structure of the target protein was obtained from the RCSB PDB and AlphaFold3, and crystallographic waters were removed in PyMOL (v3.1.1) via command-line editing. Receptors and ligands were prepared with AutoDock (4.1.0) tools to assign rotatable bonds and partial charges, then exported as PDB files. Docking simulations were performed with AutoDock Vina (1.2.0); the lowest binding energy pose was selected and visualized as a composite ligand–protein complex in PyMOL.

### 4.9. Western Blot

Hepatic protein extracts were prepared using ice-cold RIPA buffer (P0013B, Beyotime, Shanghai, China), supplemented with protease inhibitor cocktails (HY-K0010, MCE, South Brunswick Township, New Jersey, USA). Protein concentrations were determined by BCA assay (PC0020, Solarbio, Beijing, China), calibrated against BSA standards. Aliquots of 50 µg were separated under reducing conditions on 6% and 10% SDS-PAGE gels (GF1800-6, GF1800-10, Genefist, Shanghai, China) at 120 V for 90 min, then transferred to 0.45 µm PVDF membranes via wet transfer. After blocking with 5% non-fat milk for 1 h, membranes were incubated overnight at 4 °C with primary antibodies (Table S3), washed three times with TBST, and incubated with HRP-conjugated goat anti-rabbit IgG (1:5000, ab6721, Abcam, Cambridge, UK) for 2 h at room temperature. Chemiluminescent signals were detected using ECL Basic Plus (RM00020P, Abclonal, Wuhan, China) and imaged with an Amersham Imager 600 (GE Healthcare, Chicago, IL, USA). Band intensities were quantified using ImageJ 1.53k, normalized to Tubulin-α, and expressed as fold changes relative to untreated controls.

### 4.10. Statistical Analysis

All statistical analyses were executed using SPSS Statistics 25.0 (IBM Corp., Armonk, NY, USA), GraphPad Prism 9.0 (GraphPad Software, San Diego, CA, USA), and R software (version 4.2.1). Data are presented as the mean ± standard error of the mean (SEM). To strictly prevent pseudoreplication, the replicate pen (comprising 21 hens, *n* = 6 pens per group) served as the experimental unit for all in vivo production and egg quality parameters. For downstream biochemical and omics analyses, one biologically representative hen per pen was utilized, ensuring an independent sample size of *n* = 6 per treatment. The assumptions of data normality and homoscedasticity were verified using the Shapiro–Wilk test and Levene’s test, respectively. Longitudinal performance traits (e.g., laying rate and egg weight) collected over the 8-week period were analyzed using a Two-way Repeated Measures Analysis of Variance (ANOVA). In this model, “Treatment” (CON vs. SIL) served as the main effect and “Time” (Weeks 1–8) as the repeated measure. Where significant Time × Treatment interactions were detected, Sidak’s multiple comparisons test was employed to pinpoint differences at specific time points while controlling the family-wise error rate. For single-point parametric data (e.g., terminal biochemical indices and liver lipid contents), statistical differences between the two groups were evaluated using an independent, two-tailed Student’s *t*-test. Non-parametric data or data violating homoscedasticity assumptions were evaluated using the Mann–Whitney U test. Multi-Omics Data Integration: To rigorously control the risk of false positives (multiplicity) in high-dimensional datasets, Benjamini–Hochberg False Discovery Rate (FDR) correction was systematically applied. Metabolomics: Differential metabolites were identified by integrating multivariate Orthogonal Partial Least Squares-Discriminant Analysis (OPLS-DA) with univariate statistics, defined by a variable importance in projection (VIP) score > 1, |Fold Change| > 2, and an FDR-adjusted *p* < 0.05. Transcriptomics: Differential expression analysis was performed utilizing the DESeq2 R package (version 1.30.1), employing empirical Bayes shrinkage for dispersion estimation. Differentially expressed genes (DEGs) were robustly filtered based on an absolute log_2_Fold Change > 1 and an FDR-adjusted *p* < 0.05. Correlation: Associations between apparent phenotypes, key target proteins, and differential metabolites were mapped using Spearman’s rank correlation coefficient, with significant network edges defined at |r| > 0.70 and *p* < 0.05. For all analyses, statistical significance was declared at *p* < 0.05, and high significance at *p* < 0.01.

## 5. Conclusions

In summary, dietary silibinin (100 mg/kg) mitigates heat stress-induced hepatic lipid disruption and performance declines in laying hens. In a 8-week trial (THI > 73), silibinin increased laying rate and egg quality, enhanced antioxidant capacity, and reduced hepatic steatosis, lowering triglyceride and cholesterol levels. Multi-omics revealed 11 plasma metabolites (e.g., 2-oxoglutaric acid) linked to purine metabolism and 835 hepatic genes affecting PPAR signaling and fatty acid biosynthesis. Silibinin suppressed lipogenesis (downregulating ACACA, FASN) and promoted β-oxidation (upregulating CPT1A, ACSL1), confirmed by molecular docking and Western blotting. Silibinin represents a promising antioxidant feed additive to mitigate heat stress-induced hepatic impairments and support poultry productivity under challenging environmental conditions. However, as these metabolic and regulatory effects were evaluated solely against a background of thermal stress without a normothermic comparison, future investigations should incorporate thermoneutral controls to fully delineate silibinin’s baseline physiological impacts.

## Figures and Tables

**Table 1:** Protein–ligand molecular interaction results.

Protein Name	UniProt	Binding Energy (kcal/mol)	Cavity Volume (Å3)	Center (x, y, z)	Docking Size (x, y, z)	Residues	Distance (Å)
CPT1A	A0A8V1ABZ2	−9.7	5760	−6, −1, 3	26, 32, 26	Glu676, Trp682	2.57
ACSL1	A0A8V0XZI5	−8.0	20,706	5, 7, −6	35, 35, 35	Ser593, Arg683	3.23
ACACA	P11029	−8.8	2188	−52, −13, 72	26, 26, 26	His211, Asn126	2.96
FASN	P12276-2	−8.7	17,771	14, 23, −13	35, 35, 35	Arg1881, Arg1107	3.06
SCD	A0A8V0ZHE5	−8.0	3890	−7, 5, −8	26, 26, 26	Tyr529	3.00
LPL	P11602	−8.5	969	11, 5, −13	26, 26, 26	Gln60, Phe41	2.90
ACOX1	A0A8V1AQ25	−8.4	3346	−5, −1, 13	26, 26, 35	Asp615, Arg97	2.72

Molecular docking results representing predicted binding affinities and interactions.

## Data Availability

All data generated or analyzed during this study are included in the manuscript. For any additional information or requests, please contact the corresponding author.

## Supplementary Materials

The following supporting information can be downloaded at: https://www.mdpi.com/article/10.3390/ijms27104267/s1.
