# Supplementary material for: Integrative Multi-Omics Reveal Silibinin Alleviates Heat Stress-Driven Hepatic Lipid Disruption in Laying Hens
Source: Int J Mol Sci. 2026 May 11;27(10):4267. doi: 10.3390/ijms27104267 (PMC13207594; doi:10.3390/ijms27104267)
Supplement: Supplementary file 1 [file ijms-27-04267-s001.zip › Supplementary materials 1.pdf]

Table S1 Composition and nutrient level of basal diet for laying hens

Ingredient	Percentage（%）	Analyzed nutrient	Content
Corn	62.00	Metabolizable energy (Mkal/kg)	2.61
Soybean meal	24.00	Crude protein (%)	16.02
Limestone	9.00	Lysine (%)	0.74
Premix	4.50	Methionine (%)	0.38
Soybean oil	0.50	Available phosphorus (%)	0.31
		Total phosphorus (%)	0.51
		Threonine (%)	0.64
		Arginine (%)	0.99
		Calcium (%)	3.87

Note: (1) The premix consists of 1% stone powder, 1.2% calcium hydrogen phosphate, 0.12% DL-methionine, 1% composite premix, 0.3% salt (feed grade), and 0.88% carrier. (2) Nutrition level is calculated value.

Table S2 Detailed nutrition level of feed formula

Formula	AME	Cp (%)	Lys (%)	Met (%)	Ca (%)	aP (%)	Arg (%)	Thr (%)	M+C (%)	tP (%)	Percent (%)
Corn	3.24	8.7	0.24	0.18	0.02	0.12	0.39	0.30	0.38	0.27	62
Soybean meal	2.30	44.0	2.45	0.64	0.32	0.17	3.12	1.88	1.30	0.61	24
Soybean oil	8.80	0.0	0.00	0.00	0.00	0.00	0.00	0.00	0.00	0.00	0.5
Stone powder	0.00	0.0	0.00	0.00	35.00	0.00	0.00	0.00	0.00	0.00	9.00
Stone powder					35.00						1.00
Calcium hydrogen phosphate	0.00	0.0	0.00	0.00	23.20	16.50	0.00	0.00	0.00	16.50	1.20
DL Methionine	4.50	57.0	0.00	99.00	0.00	0.00	0.00	0.00	99.00	0.00	0.12
Compound premix				0.00							1
Salt				0.00							0.30
Carrier											0.88
Actual recipe	2.61	16.02	0.74	0.38	3.87	0.31	0.99	0.64	0.67	0.51	100.00

The red part is the premix composition (4.5% premix)

Table S3 Nucleotide sequences of specific primers for qPCR

Genes	Gene accession number	Forward primer (5'-3')	Reverse primer (5'-3')
ACSL1	NM_001012578	ACCGGTCTGTGCGTTGTTG	CGAGCATCCTCTTCACCCTC
CPT1A	NM_001012898	TGAGCACTCTTGGGCAGATG	TCTCCTTTGCAGTGTCCGTC
ACOX1	NM_001006205	GGAGATCGAGGCCTTAGTGA	CTGGGTGAGAAGGGTAGGGA
ACAA1	NM_001197288	AACAGCAAAGCTCGCGATTG	GCTTTGCCAAACCTTCCAGG
SCD1	NM_204890	ACCTTAGGGCTCAATGCCAC	TCCCGTGGGTTGATGTTCTG
ACACA	NM_205505	CCTGTGTTCTCTGCCTGTGT	GAAGGGCTTTTCATGCCACG
FASN	NM_205155	GCTAAGATGGCATTGCACGG	TCCATTCAGTTCCAGACGGC
LPL	NM_205282	CCGGAGCGACTCAGTTCTAC	ATCTCAGCTTCGGGATCGGA
β-actin	NM_205518	TGCTGTGTTCCCATCTATCG	TTGGTGACAATACCGTGTTCA

Table S4 List of antibodies

Antibodies	Source	Catalogue NO.	Dilution
Primary antibody			
ACACA	Abclonal	A15606	1:1000
FASN	Abclonal	A0461	1:1000
LPL	Abclonal	A16252	1:1000
SCD	Abclonal	A26246	1:1000
ACOX1	Abclonal	A8091	1:1000
CPT1A	Proteintech	15184-1-AP	1:1000
ACSL1	Proteintech	13989-1-AP	1:1000
Tubulin-α	Bioworld	BS1699	1:10000
Secondary antibody			
Goat anti-Rabbit IgG (H+L)-HRP	Bioworld	BS13278	1:10000

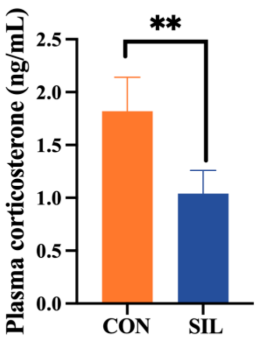

Figure S1 Plasma corticosterone
