# Supplementary material for: Integrative Multi-Omics Reveal Silibinin Alleviates Heat Stress-Driven Hepatic Lipid Disruption in Laying Hens
Source: Int J Mol Sci. 2026 May 11;27(10):4267. doi: 10.3390/ijms27104267 (PMC13207594; doi:10.3390/ijms27104267)

ACSL1

CON SIL CON SIL   CON SIL   CON SIL   CON SIL CON SIL (the same as below)

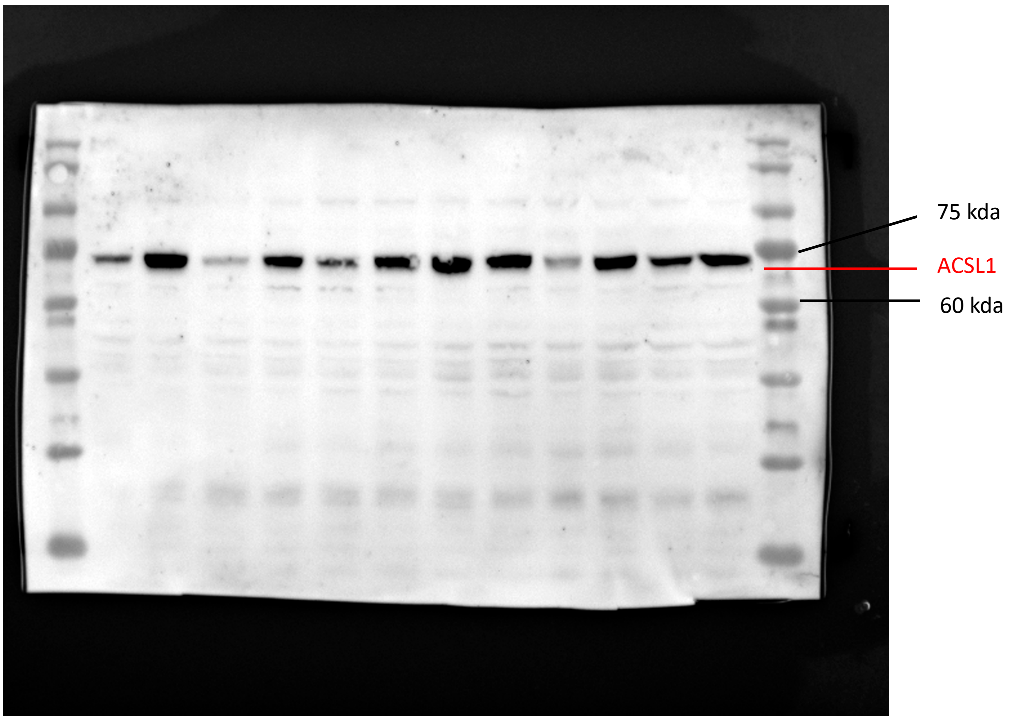

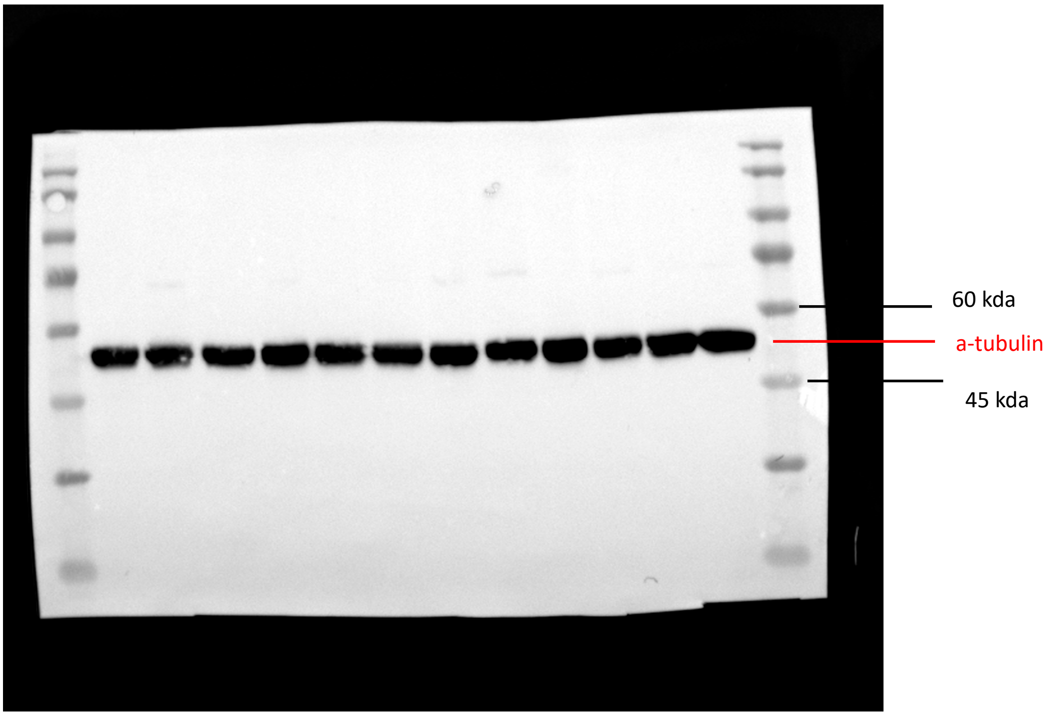

SCD1

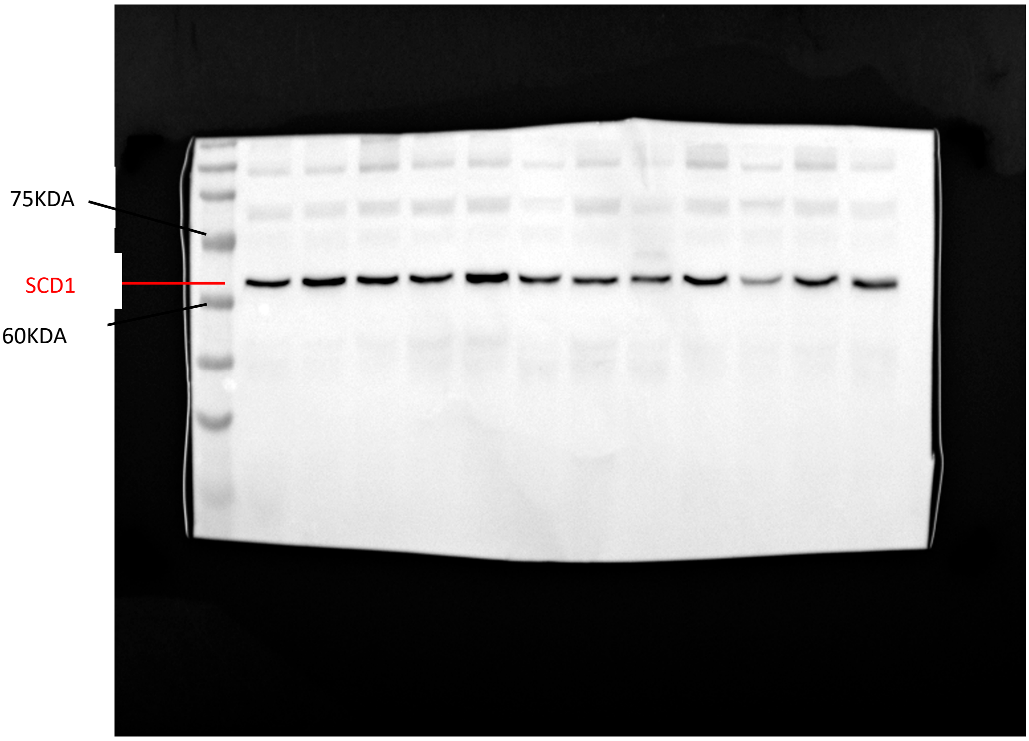

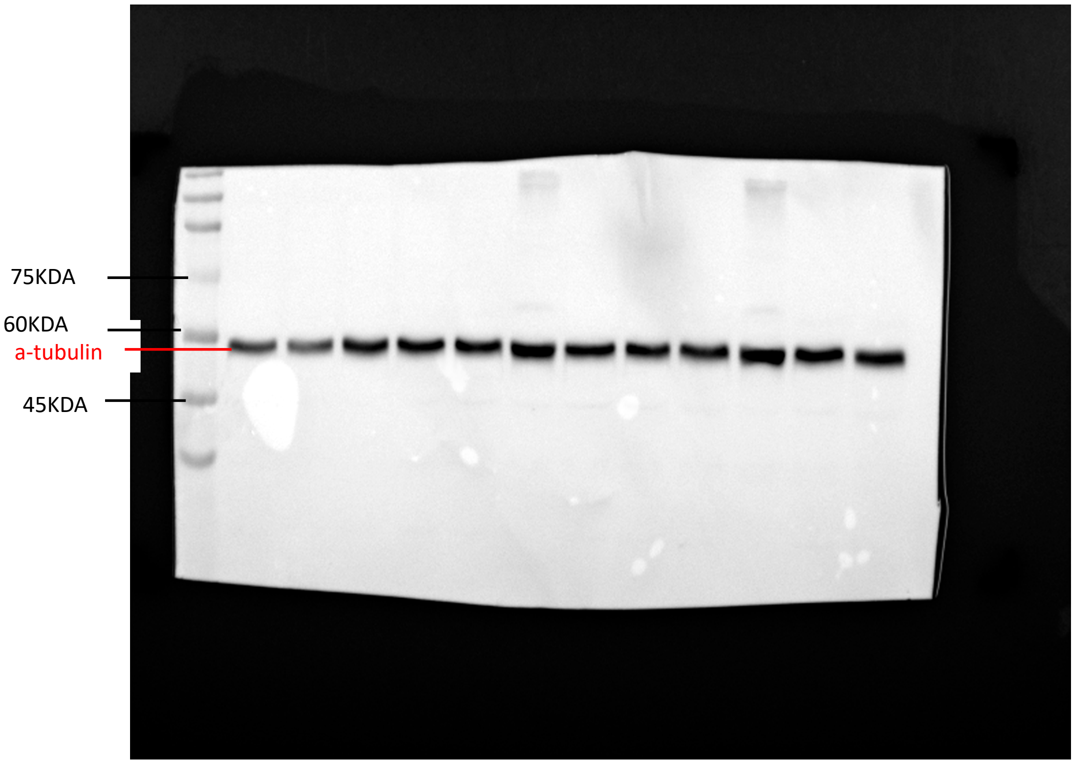

ACOX1

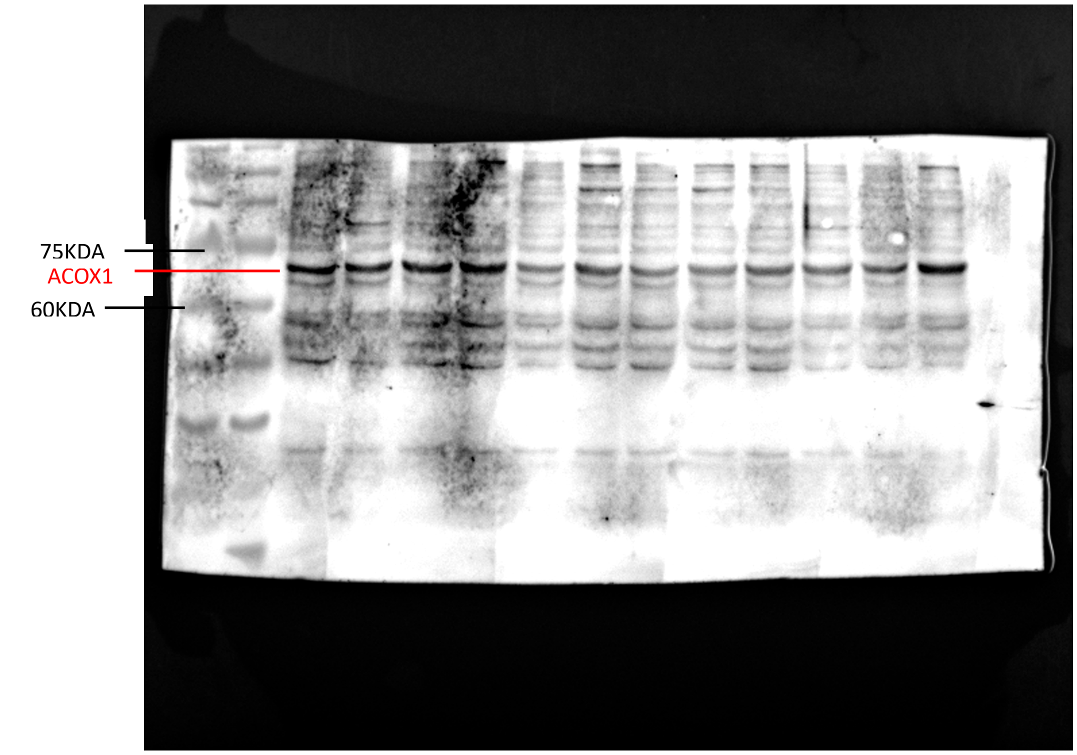

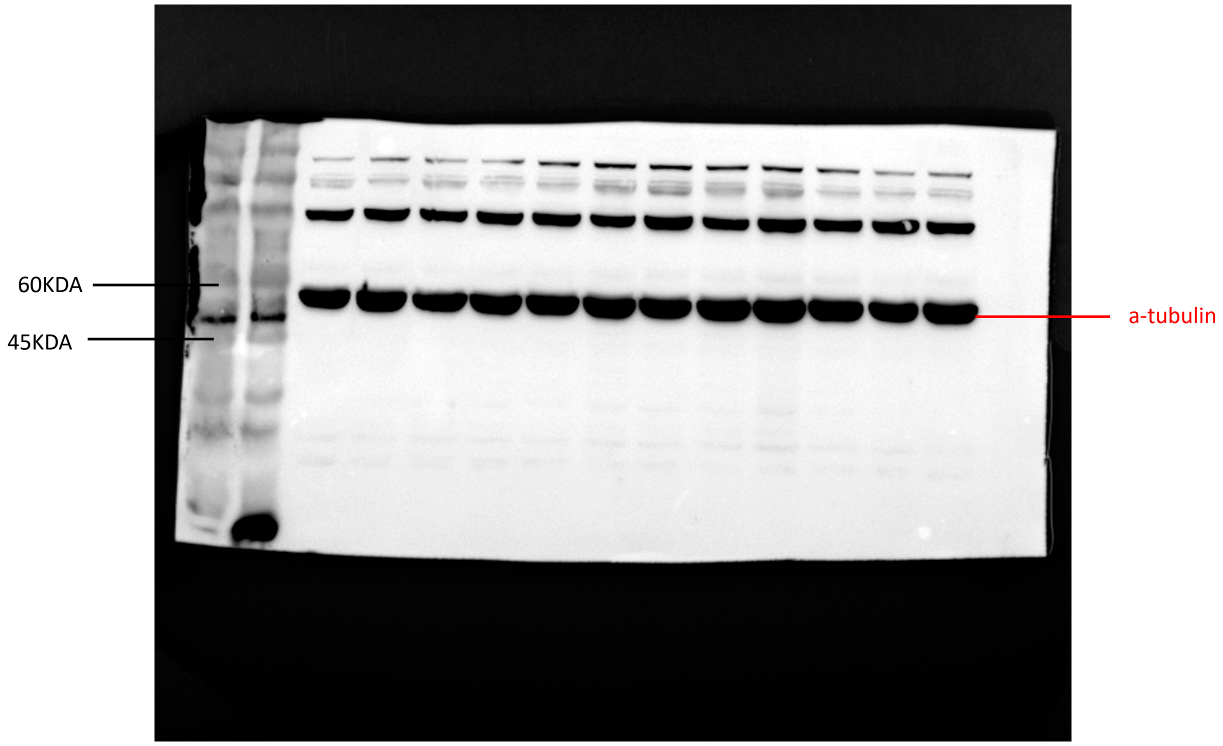

LPL

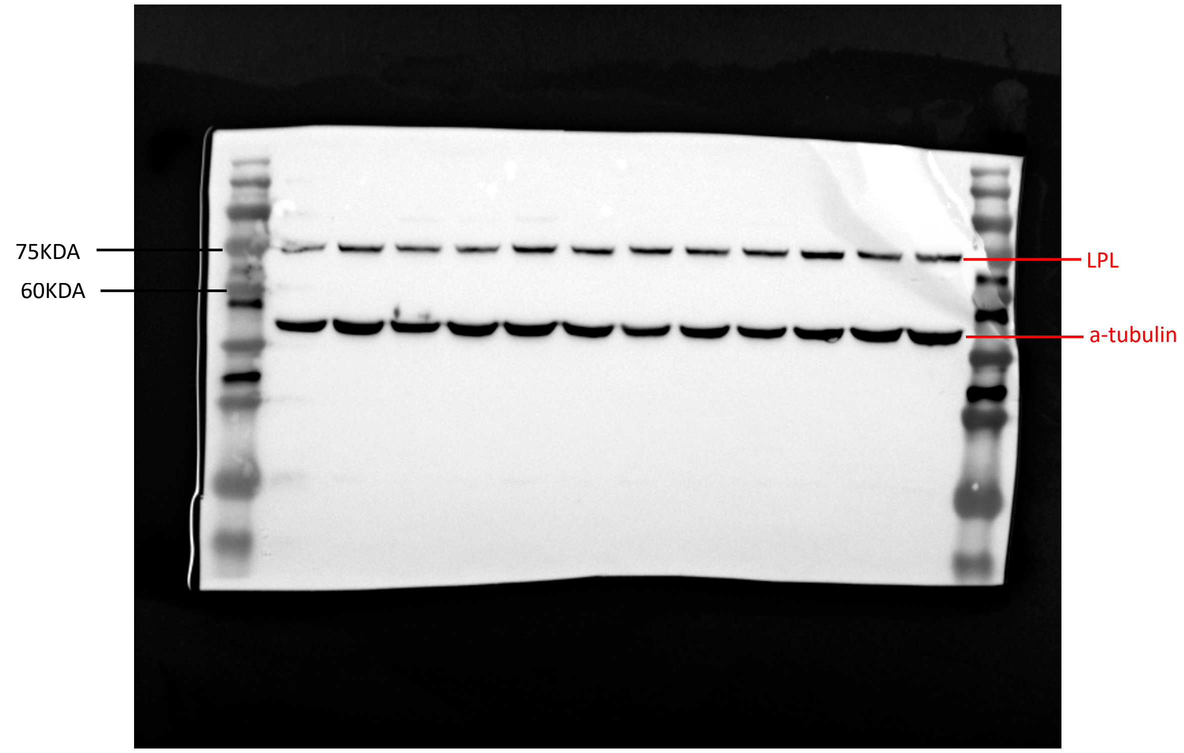

CPT1A

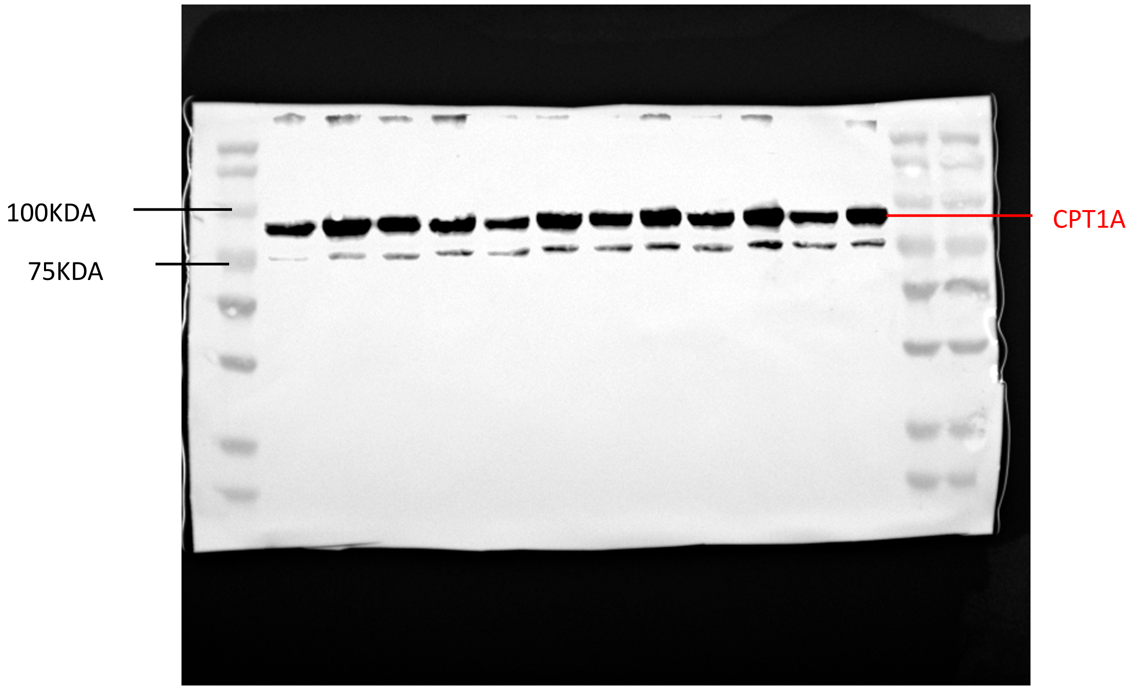

FASN

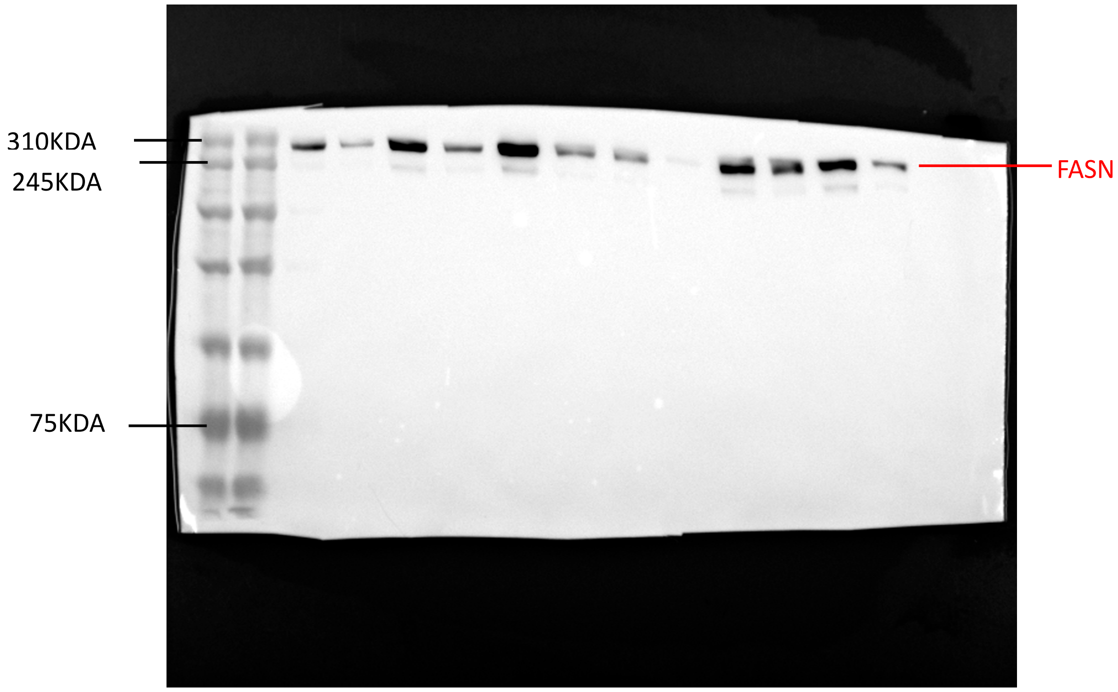

ACACA

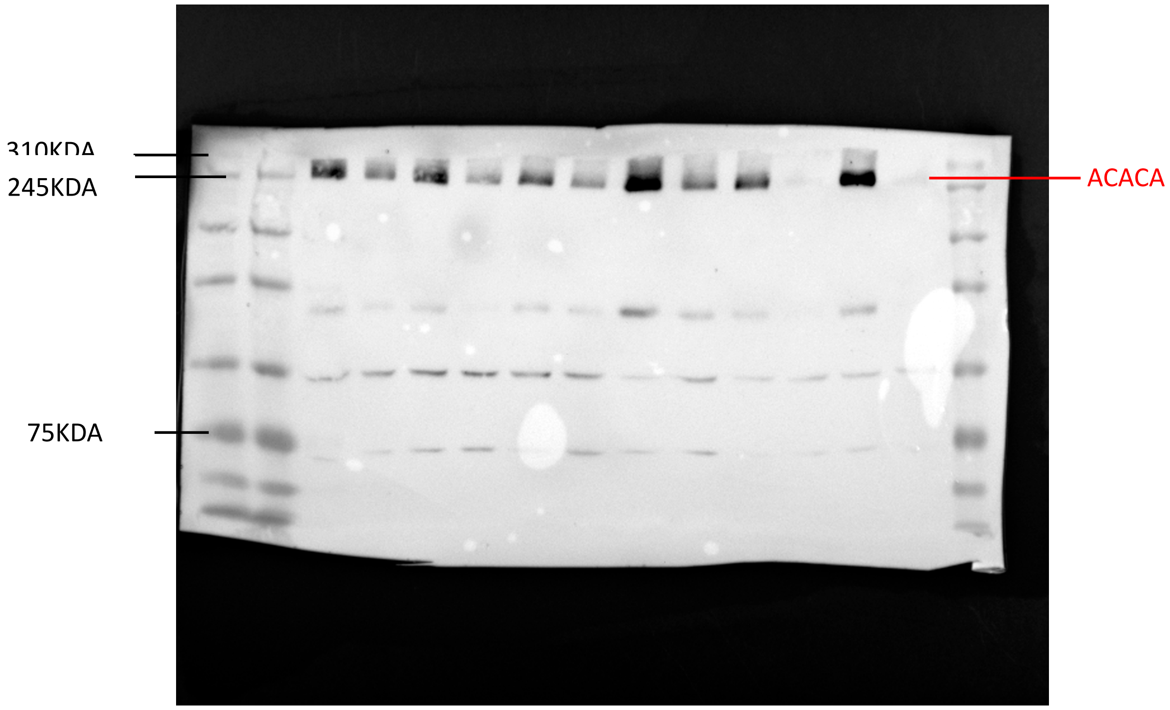

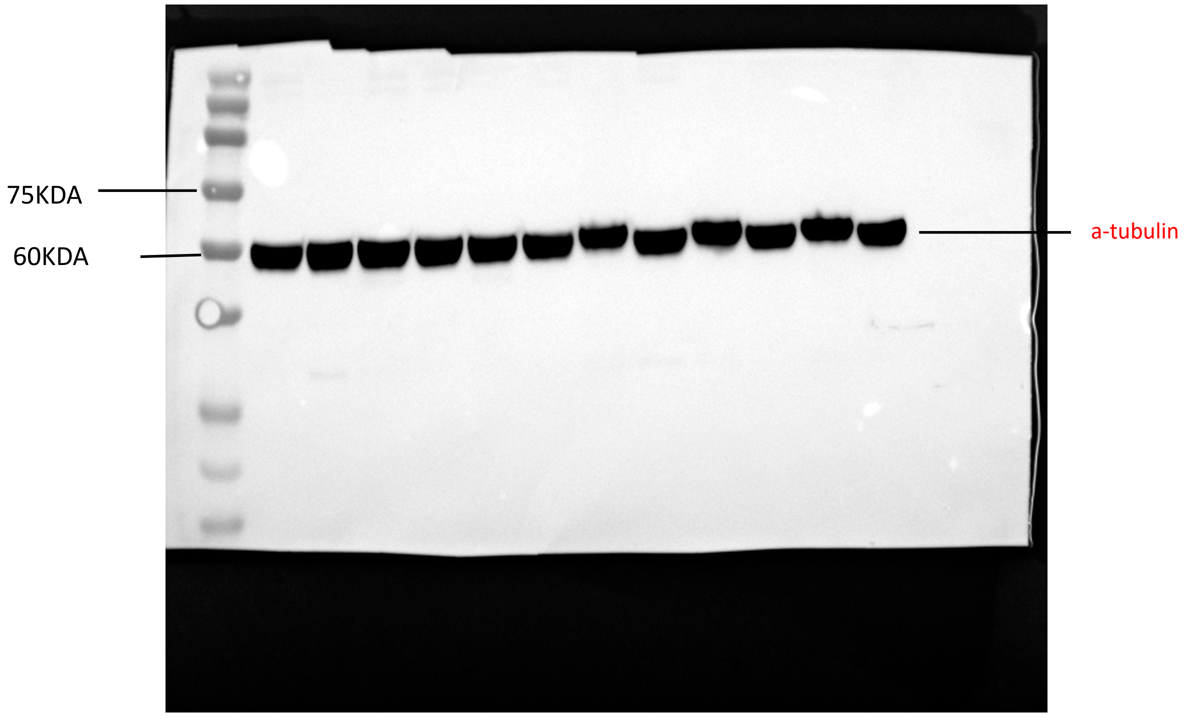

CPT1A（后补）

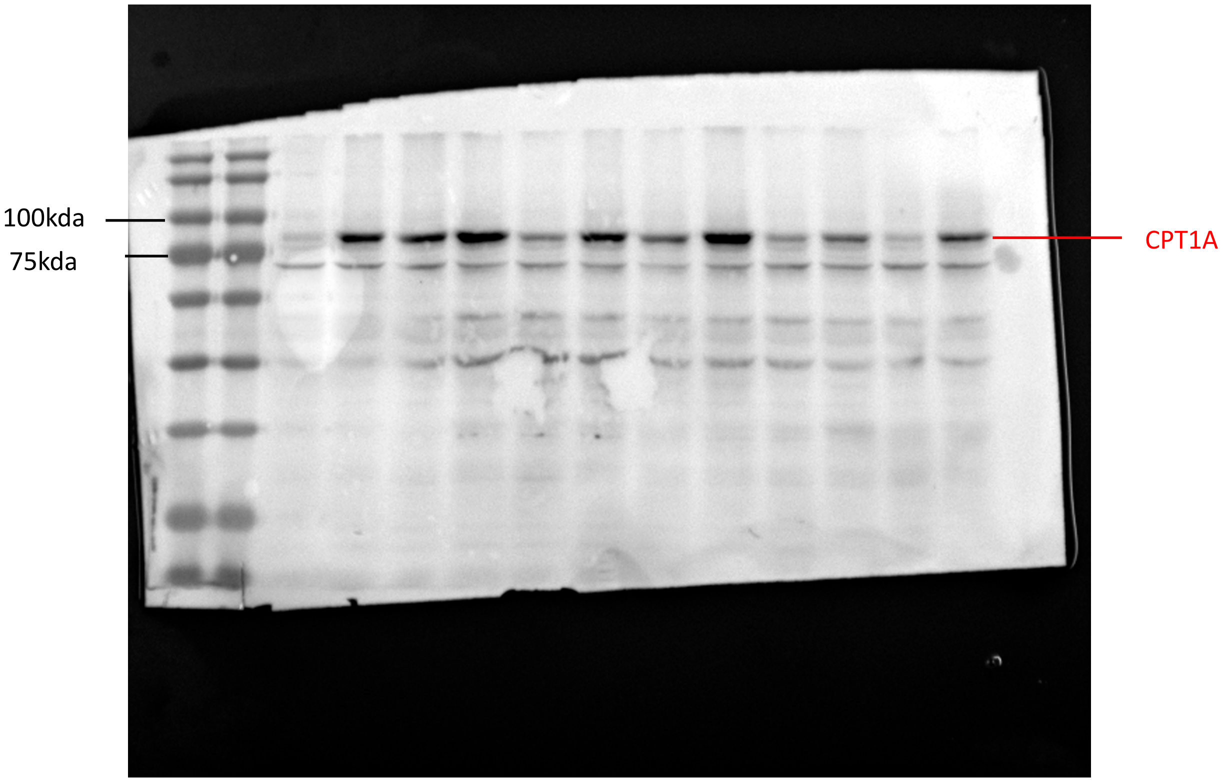

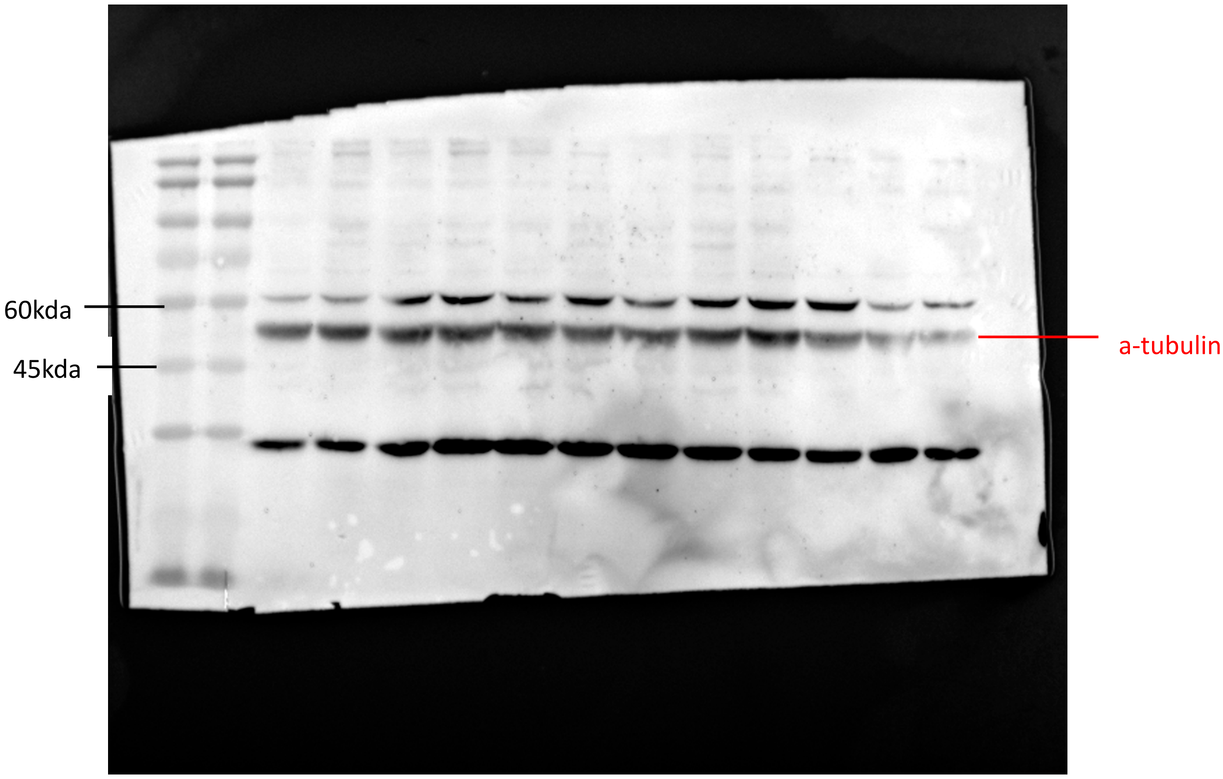

FASN（后补）

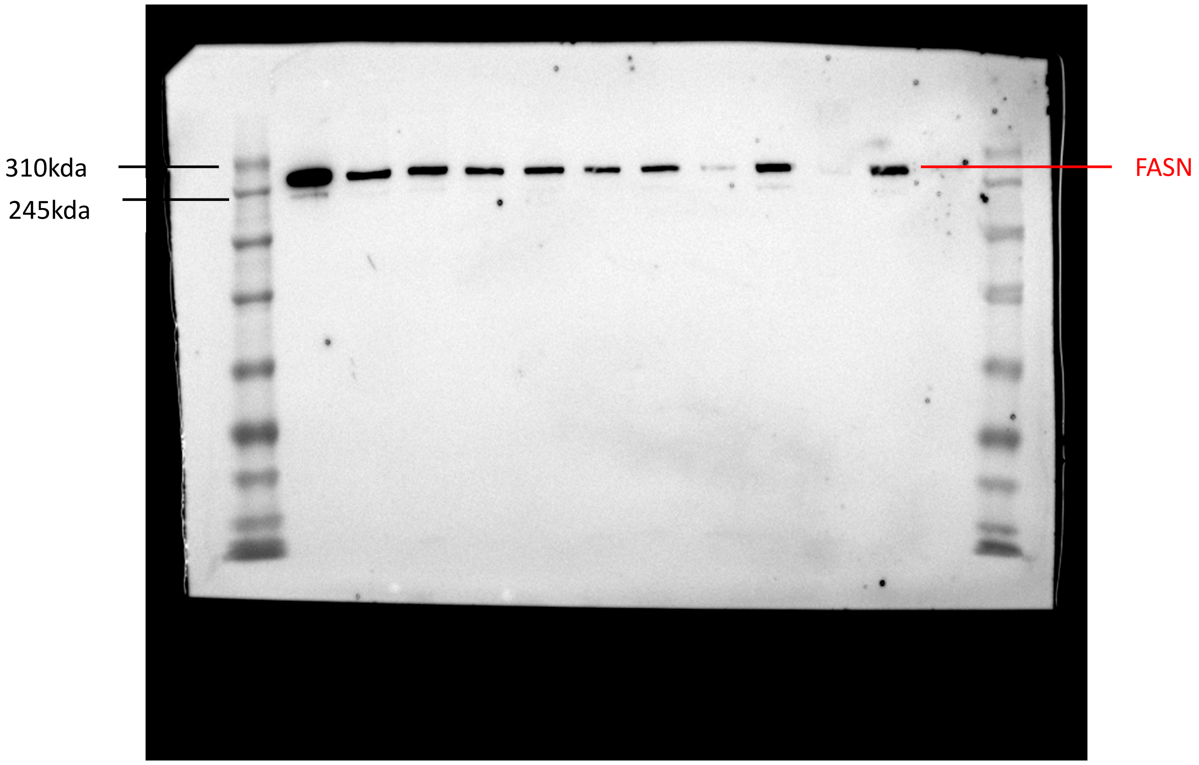

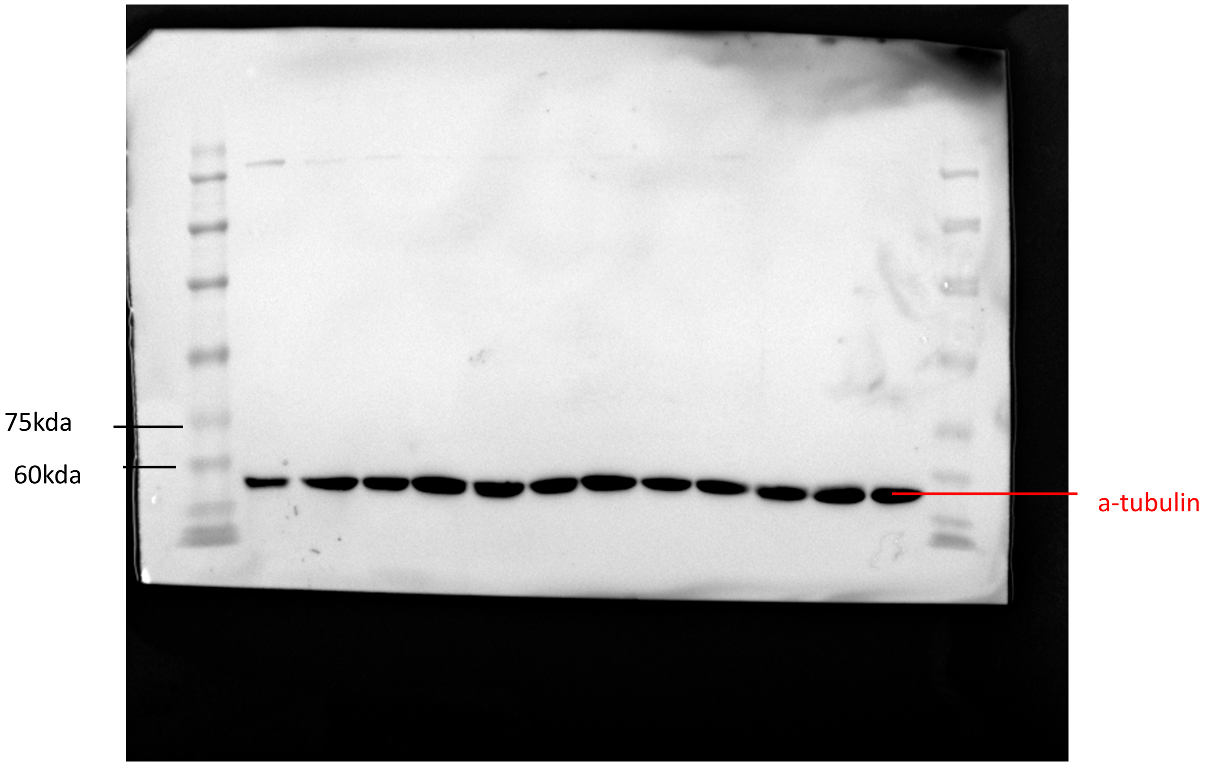

ACACA（后补）

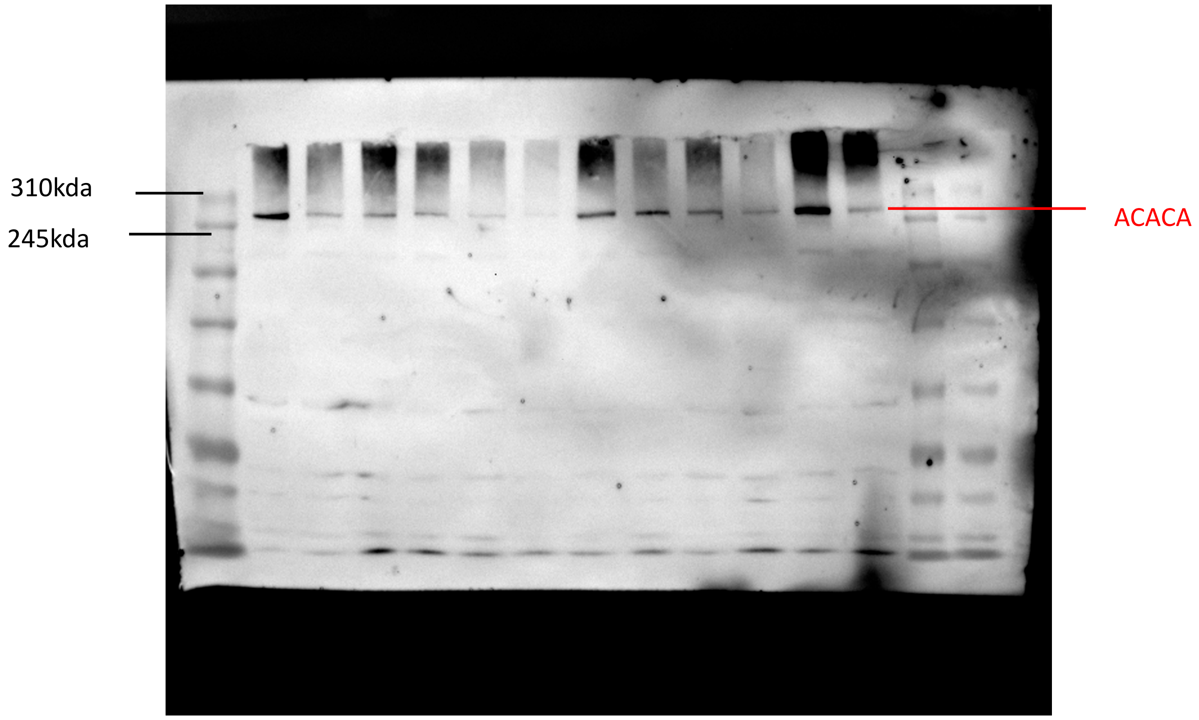

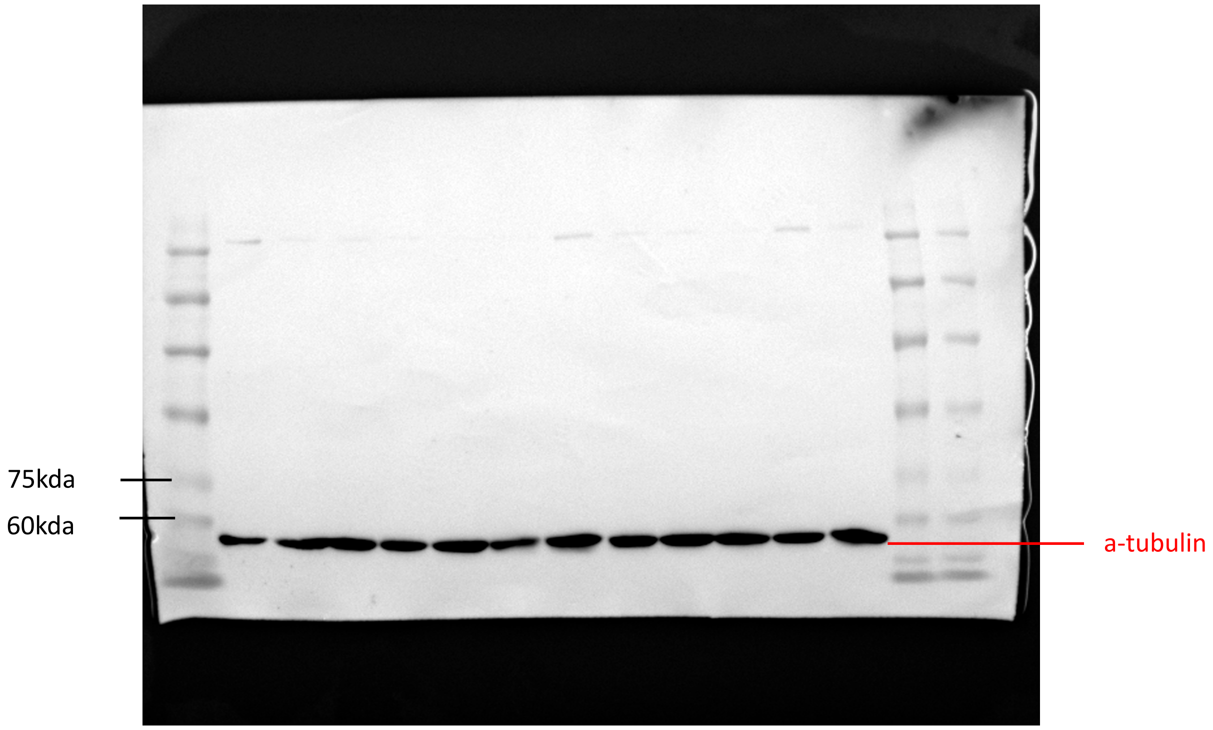

Supplement: Supplementary file 1 [file ijms-27-04267-s001.zip › Supplementary materials-2.pdf]
